# Combining multiple data sources with different biases in state‐space models for population dynamics

**DOI:** 10.1002/ece3.10154

**Published:** 2023-06-08

**Authors:** Leo Polansky, Lara Mitchell, Ken B. Newman

**Affiliations:** ^1^ U.S. Fish and Wildlife Service Sacramento California USA; ^2^ U.S. Fish and Wildlife Service Lodi California USA; ^3^ School of Mathematics University of Edinburgh Edinburgh UK; ^4^ Biomathematics and Statistics Scotland Edinburgh UK

**Keywords:** capture probability, delta smelt, estimate bias, hierarchical model, observation error, population ecology, time series

## Abstract

1. The resolution at which animal populations can be modeled can be increased when multiple datasets corresponding to different life stages are available, allowing, for example, seasonal instead of annual descriptions of dynamics. However, the abundance estimates used for model fitting can have multiple sources of error, both random and systematic, namely bias. We focus here on the consequences of, and how to address, differing and unknown observation biases when fitting models.
2. State‐space models (SSMs) separate process variation and observation error, thus providing a framework to account for different and unknown estimate biases across multiple datasets. Here we study the effects on the inference of including or excluding bias parameters for a sequential life stage population dynamics SSM using a combination of theory, simulation experiments, and an empirical example.
3. When the data, that is, abundance estimates, are unbiased, including bias parameters leads to increased imprecision compared to a model that correctly excludes bias parameters. But when observations are biased and no bias parameters are estimated, recruitment and survival processes are inaccurately estimated and estimates of process variance become biased high. These problems are substantially reduced by including bias parameters and fixing one of them at even an incorrect value. The primary inferential challenge is that models with bias parameters can show properties of being parameter redundant even when they are not in theory.
4. Combining multiple datasets into a single analysis by using bias parameters to rescale data can offer significant improvements to inference and model diagnostics. Because their estimability in practice is dataset specific and will likely require more precise estimates than might be expected from ecological datasets, we outline some strategies for characterizing process uncertainty when it is confounded by bias parameters.

## Graphical abstract

This article researches state‐space model inference about a state structured population model when abundance estimates used for model fitting contains state specific biases. A combination of theory, simulation experiments, and a case study are used to identify model identifiability and estimability, and to illustrate inference improvements and challenges when the data biases are not or are taken into account.

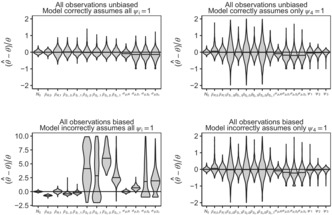

## 1 INTRODUCTION

Since the earliest uses of state‐space models (SSMs) in ecology to model fish stock population dynamics (Mendelssohn, 1988; Schnute, 1994; Sullivan, 1992) and animal movement (Anderson‐Sprecher & Ledolter, 1991), SSMs have increasingly become the near default approach for modeling wildlife population dynamics and movement. The models account for imperfect measurements of underlying true abundances or locations, the latent states, which are dynamically changing through time. Some contemporary examples of ecological SSMs and their statistical inference are provided in Auger‐Méthé et al. (2021).

Parallel to this increase in SSM usage has been an increase in the volume of data, varieties of sampling procedures, and the types of data collected. The increase is partially due to advances in technology, including numerous types of biologgers for both aquatic (Hussey et al., 2015) and terrestrial (Kays et al., 2015) organisms, camera traps (Burton et al., 2015), and remote sensing in general (Stephenson, 2019). Another reason is the growth worldwide in the number of animal population monitoring programs, often motivated by concerns over biodiversity loss (Moussy et al., 2022), as well as increased citizen science data collection. Despite the potential for multiple and possibly different sources of observation error across datasets, combining them (sometimes referred to as data integration) for use in a single analysis potentially allows the construction and analysis of more detailed and relevant quantitative models.

To take advantage of this growth in volume and types of data, SSM formulations have become increasingly ambitious and complex. The increasingly complex SSMs make model fitting more challenging, but statistical inference procedures and associated computer software have increased as well (Newman et al., 2022), so that the step of fitting SSMs is less of an obstacle. Subsequently, model formulation and practical consequences of the role that data quality places on inference have become more central. While analytical procedures are available to understand if a particular SSM is theoretically identifiable, that is, there are no redundant parameters that make inference impossible (Cole & McCrea, 2016), examples of practical difficulties in the statistical inference of SSM abound, particularly in estimating both process variation and observation error variance (Auger‐Méthé et al., 2016; Dennis et al., 2006; de Valpine and Hilborn, 2005; Knape et al., 2013). SSMs seem to have a high potential for being nearly redundant, with model parameters estimable in theory that are practically non‐identifiable with unbounded confidence intervals (Raue et al., 2009). The role of data quality in the practical estimability of SSM parameters has become a standard aspect of model inference (Auger‐Méthé et al., 2021), and in severe cases leads to extrinsic redundancy resulting in non‐identifiability of otherwise estimable parameters (Cole, 2020).

For the abundance indices used in inference about population models, data quality can involve both uncertainty related to sampling variability as well as systematic bias which is of particular interest here. By bias, we mean a constant ψ that relates the true latent abundance value N to the estimated one $\hat{N}$ used in model fitting, $\hat{N}=\mathrm{\psi N}f\left(\epsilon \right)$, where f is a function of a random variable ϵ. Such multiplicative bias commonly represents imperfect detectability or catchability of organisms during sampling (e.g., see Arreguín‐Sánchez, 1996). Model frameworks accounting for imperfect detection such as occupancy models or N‐mixture models (Royle, 2004; Sólymos et al., 2012) can potentially inform the ψ value. However, sample design requirements (e.g., multiple samples per sample location), covariate‐dependent abundances and observation detection errors, and estimation difficulties when latent states are determined by dynamic processes (Welsh et al., 2013), can preclude their use. When interest is in estimating determinants of temporally dynamic processes using available long‐term data, including a bias parameter (ψ) as part of the observation model of population dynamics SSM offers a possible option for integration of multiple abundances indices with different biases (Polansky et al., 2021).

Here we are interested in inference about parameters of a SSM that describes sequential within cohort life stage abundances linked by covariate‐dependent survival and recruitment rates, described in Section 2. General strategies for model fitting and inference are provided in Section 3. Analytical methods are used to understand requirements for model identifiability and related implications of the assumptions needed to make the model identifiable in Section 4.1. As identifiability requires vital rate processes to depend on covariates that are included in the model, we consider practical estimability in Section 4.2 under two types of data differing in their model information content. The implications of fixing model bias parameters correctly or incorrectly when data used for inference does or does not have bias is studied in Section 5, whereas previously (Polansky et al., 2021) we always correctly specified which indices had bias to be estimated and correctly fixed the remaining bias parameters at 1. A case study is provided in Section 6 to illustrate the implications of including or excluding bias parameters on state‐process inference and model diagnostics. We discuss strategies for containing estimate bounds in near‐redundant parameters in models of the type studied here and the role of model information content.

## 2 POPULATION MODEL

State‐space models are based on two parallel time series, an unknown sequence of latent states, and an associated sequence of measurements of those states, and consist of two corresponding models, a process model for the states and an observation model for the measurements (Newman et al., 2014). The SSM that is the basis for our analysis follows the four‐stage life‐cycle model from Polansky et al. (2021), which was motivated by the life cycle of a species of fish (the case study in Section 6). The model here is slightly extended to an arbitrary number, n, of life stages, and the process and observation models also have n components. The process dynamics consist of a sequence of survival rates and recruitment where a cohort‐ and stage‐specific fraction of individuals in stages 1 to n−1 survive and advance to the next life stage, and individuals in life stage n reproduce at some rate and then die. Because individuals die after reproducing, cohorts effectively do not overlap, so that the initial stage the model begins describing the dynamics will be arbitrary (here chosen to be the last stage).

Let $N_{i,t}$ denote the abundance of life stage i at time (or cohort) t. Starting with an initial abundance of reproducing adults, $N_{n,t=0}$ in cohort t=0, the abundance update equations are:

(1) $$N_{i,t}=\left\{\begin{array}{c} \rho_{t}N_{n,t-1},\text{for}\;i=1\\ \phi_{i-1,t}N_{i-1,t},\text{for}\;1<i\leq n \end{array}\right.$$

where $\rho_{t}$ is the time‐specific recruitment rate and $\phi_{i,t}$ are the life stage and time‐specific survival probabilities. The recruitment rates and survival probabilities are modeled with time‐specific environmental covariates and stochasticity as

(2) $$\;\text{Recruitment}:\rho_{t}∼\mathrm{Log}-\text{Normal}\left(w_{R,t}\beta_{R},\sigma_{p,R}^{2}\right)$$

(3) $$\;\text{Survival}:\phi_{i,t}∼\text{Logit}-\text{Normal}\left(w_{i,t}\beta_{S_{i}},\sigma_{p,S_{i}}^{2}\right),1\leq i<n$$

where $\beta_{R}^{T}=\left(\beta_{R,0},\ldots ,\beta_{R,m_{R}}\right)$ is a vector of $m_{R}+1$ regression coefficients corresponding to a vector of recruitment predictor variables $x_{\cdot ,t}$ and design matrix $w_{R,t}=\left(1,x_{1,t},\ldots ,x_{m_{R},t}\right)$, and $\sigma_{p,R}^{2}$ is the recruitment process variance on the log scale. The terms in the survival function (equation 3) are defined analogously. We will refer to the $\beta_{\cdot ,0}$ as “intercept” parameters and $\beta_{\cdot ,1},\beta_{\cdot ,2},...$ as “slope” parameters, even though on the untransformed scale the vital rate curves are not linear. Alternative formulations for how $\rho_{t}$ and $\phi_{i,t}$ are represented for this type of model were given by Maunder and Deriso (2011) and Smith et al. (2021). The quantity $N_{n,0}$ may be treated as a parameter.

The observation models for the estimated abundance index values $Y_{i,t}$ are

(4) $$Y_{i,t}∼\mathrm{Log}-\text{Normal}\left(\log\left(\psi_{i}N_{i,t}\right)-\frac{\sigma_{o,i,t}^{2}}{2},\sigma_{o,i,t}^{2}\right)$$

where $\psi_{i}$ is the bias parameter and $\sigma_{o,i,t}^{2}$ is the observation error variance. Here, we assume that for each life stage there is just one survey, hence the matching i subscript for $\psi_{i}$ and $N_{i,t}$. Variations on this formulation include (i) multiple indices for the same life stage with differing biases (Conn, 2010), (ii) in‐common biases for two or more surveys (Section 6), and (iii) the addition of a bias parameter $\psi_{0}$ for the initial observation $Y_{n,0}$ (Appendix S1: Section A).

## 3 DATA SIMULATION AND MODEL FITTING

As confounding between process variance and observation error parameters is a common and well‐documented problem (Auger‐Méthé et al., 2016; Dennis et al., 2006; de Valpine and Hilborn, 2005; Hyun and Kim, 2022), and to keep the focus on the effects of systematically biased observations, observation variances were treated as known values. Thus, the parameter vector is

$$\theta =\left\{N_{n,0},\beta_{R}^{T},\sigma_{p,R},\beta_{S_{1}}^{T},\sigma_{p,S_{1}},...,\beta_{S_{n-1}}^{T},\sigma_{p,S_{n-1}},\psi_{1},...,\psi_{n}\right\}.$$

Here, we treat $N_{n,0}$ as a fixed effect parameter (treating it as a random effect merely shifts confounding problems to the hyperparameters if they are estimated and risks assuming away sources of estimability problems that emerge in practice if the hyperparameters are a priori set).

Data simulation for simulation experiments and fitting of simulated and case study data were done within R (R Core Team, 2022). Parameter values used in simulating synthetic data used for studying practical estimability (Section 4.2) and the effects of ignoring bias or otherwise specifying it incorrectly (Section 5) are shown in Table 1. Guided by the specifics of the case study in Section 6, data were simulated for a population with n=4 life stages over 20 cohorts (*t* = 1,…,20) resulting in 80 time steps given the initialization at $N_{n,0}$.

**TABLE 1:** Parameters used for simulating data and priors of the Bayesian model. Data simulated without bias has $\psi_{i}=1$ for all i. Normal priors are specified in terms of a mean and precision $\tau =1/\sigma^{2}$. Survival‐related parameters have $\tau =3\left(m+1\right)/\pi^{2}$ where m is the number of covariates in the corresponding vital rate. Parameters not estimated were fixed at their true values during model fitting.

Parameter	Data information		Prior
	Low	High	
$\log\left(N_{n,0}\right)$	13.82	13.82	Uniform(9,15)
$\beta_{R,0}$	1.00	1.75	Normal(0,1)
$\beta_{R,1}$	−0.50	−1.00	Normal(0,1)
$\beta_{S_{1},0}$	1.00	1.00	Normal(0,0.61)
$\beta_{S_{1},1}$	1.30	2.00	Normal(0,0.61)
$\beta_{S_{2},0}$	1.00	1.00	Normal(0,0.61)
$\beta_{S_{2},1}$	1.30	2.00	Normal(0,0.61)
$\beta_{S_{3},0}$	1.00	1.00	Normal(0,0.61)
$\beta_{S_{3},1}$	1.30	2.00	Normal(0,0.61)
$\sigma_{p,R}$	0.50	0.25	Exp(1)
$\sigma_{p,S_{1}}$	0.50	0.25	Exp(1)
$\sigma_{p,S_{2}}$	0.50	0.25	Exp(1)
$\sigma_{p,S_{3}}$	0.50	0.25	Exp(1)
$\sigma_{o,1}$	0.10	0.05	Not estimated
$\sigma_{o,2}$	0.10	0.05	Not estimated
$\sigma_{o,3}$	0.10	0.05	Not estimated
$\sigma_{o,4}$	0.10	0.05	Not estimated
$\psi_{1}$	0.20	0.20	Exp(1)
$\psi_{2}$	0.10	0.10	Exp(1)
$\psi_{3}$	0.40	0.40	Exp(1)
$\psi_{4}$	0.50	0.50	Exp(1)

We used both frequentist and Bayesian‐based inference in this research. Frequentist analyses were carried out using the package TMB (Template Model Builder, Kristensen et al. (2016)) which uses Laplace approximation of random effects contributions to the likelihood in calculating maximum likelihood estimates (MLEs), ${\hat{\theta }}_{\mathrm{MLE}}$, estimates of the variance–covariance matrix, ${\hat{\sum }}_{\mathrm{MLE}}$, profile and joint profile likelihoods (Severini, 2000), and one‐step ahead prediction residuals (Thygesen et al., 2017). Bayesian analyses were done with JAGS (Plummer, 2003) using the R2jags package (Su & Yajima, 2021). Posterior samples were based on the last of 25,000 samples of 10 chains each of length 150,000, all of which had Gelman‐Rubin statistics of <1.1. Bayesian model priors are shown in Table 1 and were chosen in part using Newman (2003) to be uninformative on the natural scale.

Frequentist modeling advantages useful here include (i) computational speed needed to carry out the simulation experiments in Section 5, and (ii) ease of comparing and diagnosing models. Potential disadvantages include (i) the use of approximate likelihoods, (ii) approximated variance–covariance matrices that incorrectly obscured unestimable parameters revealed only by investigation of joint profile likelihoods. Bayesian modeling advantages relevant here include (i) the use of the exact likelihood, and (ii) the relative ease of investigating estimation problems in practice across the many possible pairs of parameters by plotting joint posteriors. Disadvantages include (i) the impossibility of specifying a truly uninformative prior (Lele, 2020), and (ii) computation time, which would have precluded the analysis reported on in Section 5.

Lack of estimability in practice was identified using profile and joint profile log‐likelihoods, or joint posterior plots. Log‐likelihoods $\ell \left(\theta_{0}\right)$ (Hilborn & Mangel, 1997), where $\theta_{0}\in \theta$ and $\ell \left(\theta_{0}\right)$ is the log‐likelihood maximized in the remaining parameters not in $\theta_{0}$, were plotted. Relatively flat surfaces near the MLEs or correlation in joint posteriors can indicate estimability problems.

## 4 IDENTIFIABILITY AND ESTIMABILITY

### 4.1 Theoretical identifiability

The role of covariates for separating out confounded parameters, including the notion that this does not automatically resolve model identifiability problems, has been discussed in Chapter 5.1 of Cole (2020). In the absence of covariate‐dependent bias parameters, an intuitive argument as to why model identifiability requires at a minimum that either the initial abundance (viewed as a parameter), or at least one of the bias parameters, is fixed (see also Conn, 2010), is the following. Without loss of generality, assume the abundance for life stage n at *t* = 0, $N_{n,0}$, is a fixed effect parameter (treating it as a random effect merely shifts confounding problems to the hyperparameters). Let u be the set of latent, time‐varying, vital rates, $u=\left\{\rho_{1},\phi_{1,1},\phi_{1,2},...,\phi_{n-1,1},\rho_{2},...\right\}$. Conditional on u, all latent abundances can be expressed as a function of $N_{n,0}$, $N_{i,t}=N_{n,0}\prod_{j=1}^{n\left(t-1\right)+i}u_{j}$ because $\left\{N_{n,0},N_{1,1},N_{2,1},...\right\}$ can be written as $\left\{N_{n,0},N_{n,0}\rho_{1},N_{n,0}\rho_{1}\phi_{1,1},...\right\}$. For each of the observation models given by equation 4, $N_{n,0}$ and the $\psi_{i}$ parameters always occur as products. Thus, a likelihood constructed with no information on $\psi_{i}$ can be constant for different value combinations of the parameters $N_{n,0}$ and $\psi_{i}$ so long as the products $N_{n,0}\psi_{i}$ remain unchanged.

The symbolic differentiation methodology described by Catchpole and Morgan (1997) and Cole (2020) and extended to state‐space models by Cole and McCrea (2016) was used to establish further conditions for model identifiability in Appendix S1. The main difference between Appendix S1: Section A and Polansky et al. (2021) is that here we establish model identifiability for arbitrary n using analytical methods (not software) for computing derivatives, along with applications of the Extension Theorem (Section 3.2.6.1 of Cole, 2020) to generalize results. This shows that model identifiability is achieved if (i) one bias parameter $\psi_{j}$ is fixed, similar to Conn (2010), and (ii) vital rates are modeled as functions of relevant covariates. Similar results hold if the initial abundance parameter is fixed in place of bias parameter $\psi_{j}$.

Although symbolic differentiation provides insight into model identifiability, it does not reveal the consequences of fixing one bias parameter at the wrong value to remove redundancy. To describe the relationships between the true biases ($\psi_{i}$) and their estimates (${\hat{\psi }}_{i}$), we examined the complete data log‐likelihood to identify sets of parameters that appear only as products (details in Appendix S1: Section B). Choose life stage j as a reference stage for which the observation bias parameter $\psi_{j}$ is fixed at a (possibly incorrect) value denoted $\psi_{\mathrm{ref}-j}$. Then the initial (adult) abundance estimate is given by ${\hat{N}}_{n,0|\psi_{\mathrm{ref}-j}}\approx N_{n,0}\psi_{j}/\psi_{\mathrm{ref}-j}$ and, correspondingly, the bias estimates are given by ${\hat{\psi }}_{i|\psi_{\mathrm{ref}-j}}\approx \psi_{i}\psi_{\mathrm{ref}-j}/\psi_{j}$ for i≠j. Because the estimate of each $\psi_{i}$ is of the true value scaled by the same factor $\psi_{\mathrm{ref}-j}/\psi_{j}$, ratios of these estimates track ratios of the true values, that is, ${\hat{\psi }}_{k}/{\hat{\psi }}_{i}\approx \psi_{k}/\psi_{i}$. Furthermore, the estimates of the state process parameters; i.e. slope, intercept, and process variance estimates, are unaffected by the choice of reference stage j and the value of $\psi_{\mathrm{ref}-j}$.

### 4.2 Practical estimability

As identifiability in the model of interest here depends in particular on the inclusion of covariates in the vital rate process, and keeping in mind general SSM inference challenges of near redundancy discussed previously, we used two simulated and contrasting datasets to identify inference abilities and pitfalls in practice. The first dataset had “high information conent” in the sense that slopes of the recruitment and survival curves (equations 2 and 3) controlled by the $\beta_{\cdot ,1}$ parameters define a stronger change in the vital rate curve as a function of the covariate, and process variation and observation error variances are small, relative to the “low information content” dataset. Abundance indices were biased in both datasets for all life stages, while in the fitted models the fixed bias parameter was (incorrectly) set at $\psi_{4}=1$.

**FIGURE 1:**
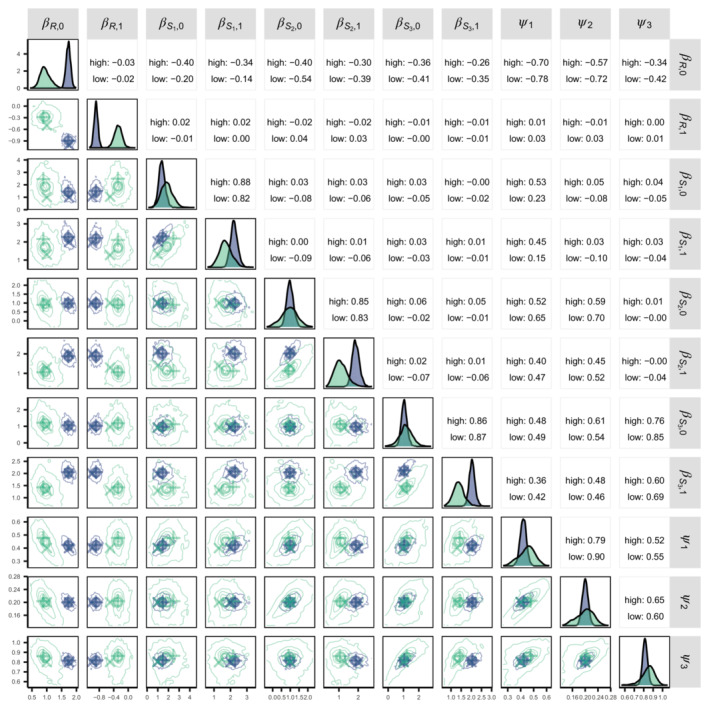
Parameter inference comparisons between fits to data with either high (purple) or low (green) information content used in the practical estimability investigation. Diagonal panels show marginal parameter posterior distributions. Off diagonal panels show joint posterior distribution contours, Bayesian posterior means (∘), MLEs (+), and true or adjusted true values (×). Adjusted true values are ${\psi_{i}}^{*}\psi_{\mathrm{ref}-j}/\psi_{j}=2\psi_{i}$ (the reference bias parameter is $\psi_{4,\mathrm{ref}}=1$ but the true value is $\psi_{4}=0.5$, see Section 4.1). Upper diagonal panels show joint posterior Pearson correlations. Initial abundance and process variance parameter results have been omitted for visual clarity. *X*‐axis limits are set by column and include the diagonal panel. *Y*‐axis limits are set by row and exclude the diagonal panel.

The state process and bias parameter MLEs and Bayesian posteriors are shown in Figure 1. For both high and low data information scenarios, MLEs and Bayesian posterior means are similar and not far from the true values. Joint posterior correlations are similar for the high and low information‐based fits and are generally greatest in absolute value between the intercept and bias parameters. Confounding between intercept (the $\beta_{\cdot ,0}$'s) and bias parameters becomes more pronounced given the low information data related to increased estimate uncertainty, illustrated by the wider marginal posterior density distributions and posterior density contours.

## 5 EFFECTS OF IGNORING OR SPECIFYING BIAS INCORRECTLY

The consequences of incorrectly assuming there is no bias, or specifying it incorrectly, are of interest as in practice whether or not abundance estimates are biased will not be known. We include the scenario where both data is unbiased and the model correctly fixes all bias parameters $\psi_{i}=1$. This provides a reference of comparison to the other more realistic scenarios of incorrectly specifying bias given the data is biased. For this portion of the analysis, we used the low information content parameters in Table 1 with n=4 over a total of 20 cohorts to simulate 1000 datasets with bias. An additional 1000 datasets without bias were simulated by fixing $\psi_{i}=1$, *i* = 1,…,4.

Two model types were fit to the simulated datasets. The first model type fixed all $\psi_{i}$ parameters at 1. This correctly matches the data generating model with respect to the bias parameters for data simulated without bias and incorrectly assumes no bias in any observations for data simulated with bias. The second model type ensures identifiability by fixing the fourth life stage bias parameter at $\psi_{\mathrm{ref},4}=1$ while estimating the remaining $\psi_{i}$. This second model type overfits data simulated without bias by including bias parameters to be estimated and incorrectly specifies the fourth life stage bias parameter for data simulated with bias which has a true value of 0.5. Thus the only fully correct model‐data pairing is the model that assumes no bias fit to data simulated without bias.

The model inference was done using TMB and summarized by MLEs compared to true values. Sometimes convergence was indicated but the Hessian calculated at the MLE location was not positive‐definite. In this case, a new dataset was simulated and the model was refit, and this process was repeated until convergence. Relative bias and mean squared error of ${\hat{N}}_{n,0}$ and ${\hat{\psi }}_{i}$ were calculated in reference to the true value of the quantity being estimated given that $\psi_{4}$ was fixed. That is, ${\hat{N}}_{0}$ is compared to $N_{0}\psi_{4}/\psi_{\mathrm{ref}‐4}$ and ${\hat{\psi }}_{i}$ is compared to $\psi_{i}\psi_{\mathrm{ref}‐4}/\psi_{4}$ for i = 1, 2, 3 (see Section 4.1).

**FIGURE 2:**
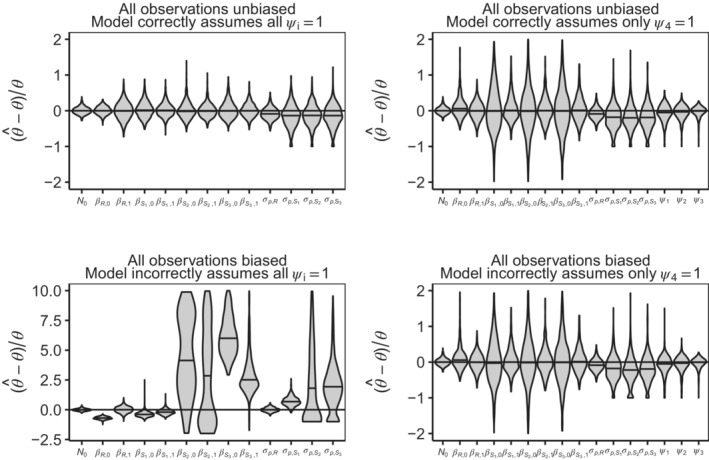
Relative biases of MLEs for simulated datasets across four combinations of data and model types. *Y*‐axis limits have been constrained for clarity and are the same in all but the bottom left panel. The top panel row has no bias in observations, and the bottom panel row has bias in all observations (see Table 1). Only the data‐model pair in the top left panel exactly match. Relative error calculations for ${\hat{N}}_{0}$ and ${\hat{\psi }}_{i}$ use adjusted true values ${N_{0}}^{*}\psi_{j}/\psi_{\mathrm{ref},j}=N_{0}/2$ and ${\psi_{i}}^{*}\psi_{\mathrm{ref}-j}/\psi_{j}=2^{*}\psi_{i}$ because the reference bias parameter is $\psi_{4,\mathrm{ref}}=1$ but the true value is $\psi_{4}=0.5$ (see Section 4.1).

Differences in MLE distributions across the four combinations of data and model type are shown in Figure 2 (see also Appendix S2: Table S2 for summary statistics). When all observations are unbiased and no bias parameters are estimated, the error is generally centered around zero. As expected, including bias parameters to be estimated in the model when the data is unbiased results in increased variance in parameter estimates (compare panels in the top row of Figure 2), reflecting increased estimate uncertainty. When all observations are unbiased and the model incorrectly assumes no bias, vital rate slope and intercept parameter estimates become relatively largely biased, and process variance parameters become positively biased (bottom left panel in Figure 2). Even with the reference bias incorrectly fixed, parameter estimate error comes centered around 0 and the variance is reduced for all parameters (bottom right panel in Figure 2).

Additional simulation study results on model fitting and parameter estimates are provided in Appendix S2. Some general remarks based on these model fits are the following. Process variance parameter estimates often converged unrealistically toward zero (Appendix S2: Table S2 and Figure S2) even though observation error variance parameters were fixed at their true values. Pairwise distributions of MLEs (Appendix S2: Figures S3–S6) mimic the patterns of the posterior sample shown in Figure 1. Taken together, these simulations and estimates indicate that parameter uncertainty includes contributions from confounding, and that process variance will be underestimated.

## 6 CASE STUDY

### 6.1 Data and models

We used the model framework described in Section 2 to fit three SSMs to data from multiple surveys of the fish species delta smelt (*Hypomesus transpacificus*). Abundance estimates and variances were obtained from Polansky et al. (2019) for n=4 life stages corresponding to spring post‐larvae ($S_{1}$), summer juveniles ($S_{2}$), fall sub‐adults ($S_{3}$), and winter reproducing adults ($S_{4}$). The estimates were for 25 cohorts from 1991 to 2015 that were sampled using trawl surveys carried out by the Interagency Ecological Program (https://wildlife.ca.gov/Conservation/Delta). Indices summarizing potential drivers of recruitment and survival (e.g., food and predator indices, abiotic environmental conditions) by life stage and cohort used as predictor variables are listed in Appendix S3: Table S3.

Abundance estimates (Appendix S3: Table S3) for stages $S_{1}$, $S_{2}$ and $S_{3}$ are labeled TMM, STN, and MWT, respectively, with the TMM survey operating only since 1995. Stage $S_{4}$ abundance estimates were based on MWT data during 1991–2001 and starting in 2002 used data from a survey labeled here as SKT. Only the TMM and SKT surveys were designed specifically for monitoring delta smelt. With the exception of SKT, which collects samples using a net pulled at the water surface, sampling is done by deploying nets obliquely through the water column. In a previous study (Polansky et al., 2021) we had assumed the TMM and SKT surveys had the same relative bias and fixed both $\psi_{\mathrm{TMM}}$ and $\psi_{\mathrm{SKT}}$ at 1. However, recent pilot gear efficiency studies (United States Fish and Wildlife Service et al., 2021) have suggested that early life stages may be more surface‐oriented than previously believed, which may affect the availability probability because TMM samples are conducted by pulling a net behind a boat which may disperse fish. Sampling biases in such surveys can result from different factors (e.g., environmental conditions, fish avoidance behavior of sample gear, a mismatch between the sample frame and overall population) and have been discussed at length elsewhere (Mitchell et al., 2019; Peterson & Barajas, 2018; Polansky et al., 2019).

The three models differed according to assumptions about bias in the abundance estimates used as observations. The first model, $m_{0}$, fixes all observation bias parameters at 1 (i.e., assumes all abundance estimates are unbiased), and serves as a null model to compare with models estimating one or more bias parameters. The second model, $m_{1}$, sets the TMM and SKT observation model bias parameters at 1 and estimates bias parameters for the STN and MWT surveys; $m_{1}$ is similar to the Bayesian SSM model fit in Polansky et al. (2021) but was revisited here to allow STN and MWT biases to be larger than 1 and allow consistent methods for model comparison. In the third model, $m_{2}$, the SKT observations were used as the reference survey by setting $\psi_{\mathrm{ref}‐\mathrm{SKT}}=1$ while all remaining bias parameters were estimated, allowing the maximal amount of uncertainty related to bias in the observations.

Models were fit using TMB. One‐step ahead prediction residuals (Thygesen et al., 2017), parameter profile likelihoods, the likelihood ratio test (Severini, 2000), and graphical examination of vital rate estimate differences between models were used to compare models. Comparisons of vital rate processes across models were made by simulating 10,000 random realizations of the models parameterized with ${\hat{\theta }}_{\mathrm{MLE}}$ for each of a set of covariate values ranging from the minimum to the maximum observed. The point estimate was taken as the mean, and lower and upper measures of uncertainty used the 2.5 and 97.5 percentiles, respectively, of these simulations. As such, the uncertainty described here is driven by process stochasticity.

Parameter estimate uncertainty will widen prediction intervals in a straightforward and relatively minor way when parameter estimates are bounded and not practically confounded (Polansky et al., 2021). Anticipating from the simulation experiments confounding in a model with as many bias parameters estimated as possible we developed the following approach to better understand uncertainty driven by this confounding for model $m_{2}$. To examine the effects of uncertainty in the intercept parameters $\beta_{R,0}$, $\beta_{S_{1},0}$, and $\beta_{S_{2},0}$ on vital rate curves, we constructed a grid of triplets $\left\{\beta_{R,0},\beta_{S_{1},0},\beta_{S_{2},0}\right\}$ over which profile likelihood parameters were estimated. The grid limits were the 95% confidence interval limits of each parameter from Table 2 when defined. The upper limit of $\beta_{R,0}$ was based on a maximum potential fecundity (100% egg survival). The egg production‐length‐based relationship described in (Damon et al., 2016) was used to compute a maximum egg production value at the average fork length of 72.06 mm, the maximum observed length in the cohorts studied here, predicts 1999.91 eggs per female. Assuming a sex ratio of 0.5, this limit is $5.82=\log\left(1999.91*0.5\right)-{\hat{\beta }}_{\mathrm{ACM}}\bar{x}$, where ${\hat{\beta }}_{\mathrm{ACM}}=0.46$ is the slope parameter for the adult food prey index and $\bar{x}$ is the maximum value of the adult prey food used in model fitting. The lower limits of the survival intercepts were set at an order of magnitude smaller than the estimated survival rate with covariates at their mean value $\text{logit}\left(1/\left(1+\exp\left(-{\hat{\beta }}_{S_{i},0}\right)\right)/10\right)$. Each grid dimension consisted of 10 equally spaced points, including the end points. Index the grid location using $\left\{i,j,k\right\}$, the profile likelihood value at location $\left\{i,j,k\right\}$ by $\ell \left(\theta_{0,i,j,k}\right)$, and the profile likelihood parameter vector by ${\hat{\theta }}_{0,i,j,k}$. Grid location $\left\{i,j,k\right\}$ was retained for inclusion in the set of vital rate curves if the profile likelihood value $\ell \left(\theta_{0,i,j,k}\right)$ was not rejected by a likelihood ratio test with reference to the unconstrained model at ${\hat{\theta }}_{\mathrm{MLE}}$ with a *p*‐value of .05.

**TABLE 2:** Delta smelt model comparison statistics (a) and select parameter estimates (b).

	$m_{0}$	$m_{1}$	$m_{2}$
(a) Maximum log‐likelihood and ΔAICc values.			
log‐likelihood	−183.24	−120.21	−111.00
ΔAICc	132.94	14.46	0

Parameter	$m_{0}$			$m_{1}$			$m_{2}$		
	MLE (SE)	LCI	UCI	MLE (SE)	LCI	UCI	MLE (SE)	LCI	UCI
(b) Maximum likelihood estimates (MLE), standard errors (SE), and 95% lower (LCI) and upper (UCI) confidence intervals using likelihood ratio tests of profile likelihoods. Entries with NA denote values not estimable. Bias parameters not estimated have entries at their set value of $\psi_{\mathrm{ref}=i}=1$. The remaining parameter estimates are reported in Appendix S3: Table S3.									
$\beta_{R,0}$	3.63 (0.17)	3.29	3.97	2.75 (0.16)	2.44	3.07	4.81 (1.09)	3.60	NA
$\beta_{S_{1},0}$	−1.37 (0.21)	−1.77	−0.90	0.00 (0.49)	−0.84	1.24	−0.88 (0.54)	NA	0.20
$\beta_{S_{2},0}$	−1.16 (0.26)	−1.68	−0.62	−0.52 (0.45)	−1.33	0.53	−2.20 (1.24)	NA	−0.76
$\beta_{S_{3},0}$	3.10 (1.07)	1.56	6.38	0.49 (0.50)	−0.31	1.88	−0.65 (0.39)	−1.32	0.44
$\psi_{\mathrm{TMM}}$	1			1			0.08 (0.09)	NA	0.31
$\psi_{\mathrm{STN}}$	1			0.44 (0.08)	0.30	0.21	0.06 (0.06)	NA	0.21
$\psi_{\mathrm{MWT}}$	1			0.18 (0.03)	0.13	0.26	0.08 (0.03)	0.04	0.15

### 6.2 Results

Model $m_{2}$ had the most support and model $m_{0}$ had the least support based on sample size corrected Akaike information criterion (AICc) (Table 2) and likelihood ratio tests. All models had significantly different maximum likelihood values from each other ($m_{0}$ and $m_{1}$ test statistic = 126.06, df = 2, *p*‐value <.01; $m_{0}$ and $m_{2}$ test statistic = 144.47, df = 3, *p*‐value <.01; $m_{1}$ and $m_{2}$ test statistic = 18.42, df = 1, *p*‐value <.01). One‐step ahead prediction residuals show that model $m_{0}$ systematically underpredicts stage 3 abundance and overpredicts stage 4 abundances starting in 2005 (Appendix S3: Figure S3). Estimated biases of surveys relative to the SKT were always <1 (Table 2). Remaining point estimates, standard errors, and profile‐based 95% confidence intervals are given in Appendix S3: Table S3.

Profile likelihoods were approximately parabolic around the maximum likelihood estimate (Appendix S3: Figure S3) with the following exceptions for model $m_{2}$: three intercept parameters ($\beta_{R,0}$, $\beta_{S_{1},0}$_,_ and $\beta_{S_{2},0}$) and two bias parameters ($\psi_{\mathrm{TMM}}$ and $\psi_{\mathrm{STN}}$). These parameters showed “ridges” in their profile likelihoods of relatively constant values over large changes of the profiled parameter. Investigation of joint profile likelihoods and variance–covariance matrices indicated confounding between these bias and intercept parameters (Appendix S3: Figure S3), and that $m_{2}$ had a somewhat geometrically complex model likelihood function.

**FIGURE 3:**
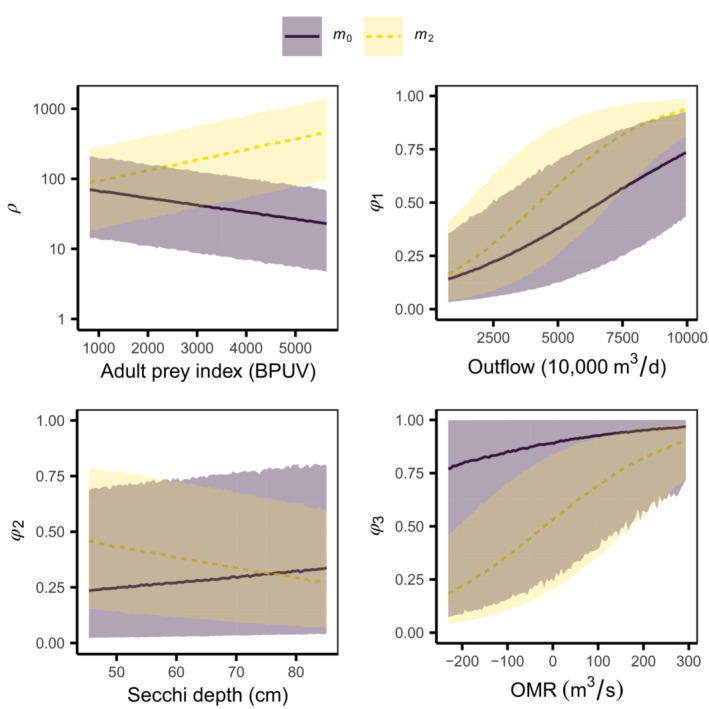
Delta smelt case study vital rate curves, recruitment, and survival for three stages, as functions of selected covariates and all other covariates fixed at their mean values, for model $m_{0}$, which assumed no bias, and model $m_{2}$, which estimated all but one bias parameter. Thick lines are mean values from simulated values given ${\hat{\theta }}_{\mathrm{MLE}}$, and shading extends between the 2.5 and 97.5 percentiles based on process stochasticity.

MLE‐point‐based vital rate prediction curves as functions of each covariate for each model are shown in Appendix S3: Figure S3. Vital rate prediction curves contrasting models $m_{0}$ and $m_{2}$ are shown in Figure 3 using the subset of covariates with the largest absolute value of the slope estimate of each vital rate from model $m_{2}$ (Appendix S3: Table S3). This subset includes an adult prey food index in biomass per unit volume (BPUV) for recruitment ρ, post‐larvae survival summer outflow for $\phi_{1}$, a measure of water clarity (Secchi depth) for juvenile survival $\phi_{2}$, and a hydrodynamic flow index (OMR) related to winter water exports for sub‐adult survival $\phi_{3}$. The most notable difference is model $m_{2}$ which shows a clear decline in survival with decreases in OMR, consistent with expectations, compared with model $m_{0}$ whose upper uncertainty limit remains near 1 across the entire range of values. Another difference is that recruitment is predicted to increase with increased food for model $m_{2}$ but decreases for model $m_{0}$.

**FIGURE 4:**
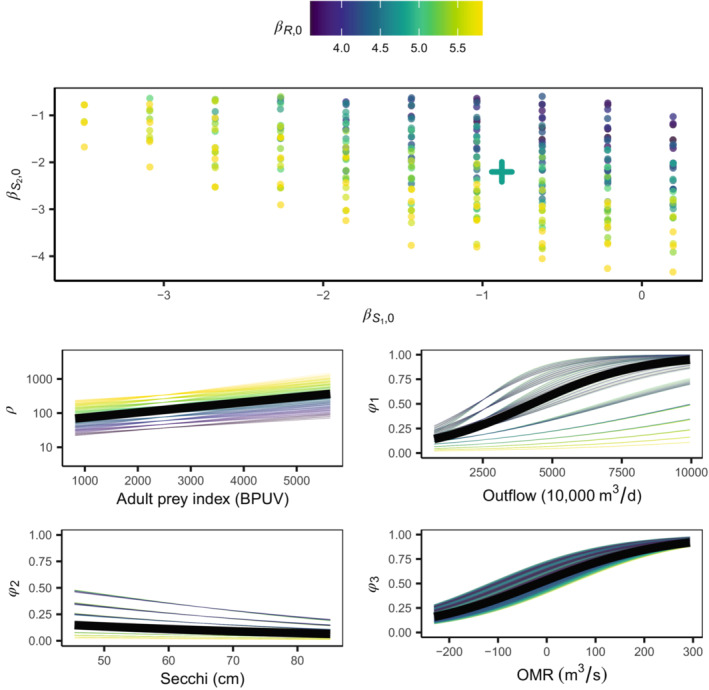
Delta smelt model $m_{2}$ vital rate curves illustrating how confounding and subsequent unbounded uncertainty in the $\beta_{R,0}$, $\beta_{S_{1},0}$, and $\beta_{S_{2},0}$, translates into uncertainty in the vital rate curves. Colors are according to the recruitment intercept value $\beta_{R,0}$. The scatterplot in the top panel shows the grid of values for which parameters were not rejected according to a likelihood ratio test and *p*‐value <.05 (dots), with the MLE denoted by a +. Corresponding vital rate curves are shown in the middle and bottom panel rows, with black lines at the MLE defined curves. Note the *y*‐axis of the recruitment panel is on a base 10 log scale.

The parameter estimate covariance matrix ${\hat{\sum }}_{\mathrm{MLE}}$ for model $m_{2}$ indicates confounding between the $\beta_{R,0}$, $\beta_{S_{1},0}$_,_ and $\beta_{S_{2},0}$ parameters relative to models $m_{0}$ and $m_{1}$ (Appendix S3: Figure S3). The implications for vital rate inference is that recruitment and either stage 1 or stage 2 survival (on the natural scale) are confounded, as illustrated in Figure 4. If the expected recruitment estimate increases as controlled by the $\beta_{R,0}$ parameter, subsequent survival rate estimates will decrease.

## 7 DISCUSSION

An underused feature of SSMs is their ability to not only account for random sample error but systematic bias as well. We found this to be an important consideration of the population model here when the abundance estimates used to fit the model have different biases. Given biased abundance observations for population modeling, omitting bias parameters from the observation model can yield qualitatively incorrect estimates of process parameters, for example, slope coefficients can change from positive to negative. If observations were unbiased, the tradeoff for including additional bias parameters was a reduction in precision, a result that is consistent with the bias‐variance tradeoff. Including estimation of bias parameters, even when the fixed value for the reference parameter $\psi_{\mathrm{ref}‐j}$ is incorrect, can improve estimates of intercept and slope coefficients linking covariates to the vital rates. For the case study, allowing bias estimation resulted in clearly improved model diagnostics and reduced uncertainty about stage 3 survival as a function of OMR, an important management consideration.

The primary drawback to including bias is that intercept and bias parameters quickly become confounded. Even the high model information content data as defined and simulated here showed parameter correlation similar to the low information content data. Emerging likelihood ridges (confounding) from low model information in practice occurs in non‐SSMs settings as well, for example, in occupancy analyses (Royle et al., 2012). Here, this suggests the population model is near‐redundant, that is, statistical estimation behaves practically like a model with redundant parameters, and bounded estimates will rely on perhaps ecologically unrealistic levels of process variation and observation error variance. These difficulties are in addition to estimating a SSM with bias factors in addition to already problematic estimation of process variance even given data that is unbiased and no bias parameters are included in the model, which seems to be a recurring issue with SSMs in general (Auger‐Méthé et al., 2016, 2021). Nevertheless, ignoring bias completely seems to be risky given the simulation and case studies presented here.

There are at least several options to help overcome the inference challenges found here when including bias parameters. One is to include an estimation of just as many bias parameters as needed to remove any especially problematic model diagnostics, such as the $m_{1}$ model of the case study. Although not the preferred model by AICc, inference about its parameters seemed more tractable for application given the parabolic and bounded profile likelihoods describing estimate uncertainty. Another option that might help overcome confounding, or at least bound the parameters, is to include additional demographic data beyond the abundance indices. We used upper bounds on recruitment derived from Damon et al. (2016) to put an upper estimate on the recruitment rates and better understand how the uncertainty between parameters shaped vital rates. This and similar information might be brought to bear formally in the model fitting step using an informed prior in a Bayesian model, but generally, this will likely involve tailored modifications to the observation and process models and are beyond the aim of the research here.

Some computational lessons gathered in this research were that frequentist and Bayesian posterior means closely agreed, even when uncertainty could be high. The use of TMB to study estimation across many simulated datasets, and to construct profile likelihoods, was invaluable. A pitfall we encountered here related to this approach was that for model $m_{2}$ the variance covariance matrix ${\hat{\sum }}_{\mathrm{MLE}}$ reflected only the relatively local curvature around the point estimate so that confounding was not well described; profile likelihoods revealed important uncertainty about intercept parameters in particular, echoing the guidelines for robust SSM inference pointed out by Auger‐Méthé et al. (2016). A Bayesian approach may be easier in practice to incorporate additional information and to capture parameter estimate uncertainty vital rate inference when this uncertainty is non‐normal or otherwise involves complicated confounding with other parameters. Although not investigated here, methods such as integrated nested Laplace approximations for Bayesian inference may offer a more efficient alternative in future studies of many datasets within a Bayesian framework is of interest than model specification and posterior sampling via JAGS used here.

One expansion of the model type considered here would be to consider non‐constant functions for the bias models. Observation biases are likely to vary as functions of covariates, space, and time (Korman and Yard, 2017; Wilberg et al., 2010 and citations therein). Unreported fits with the case study including a covariate‐controlled bias parameter indicate this is at least feasible, but work on better understanding inference in these situations is needed. Underpinning the abundance estimates used for the model fitting in the case study here is sample‐specific information, and combining, for example, zero‐inflated catch models sensu Sólymos et al. (2012) with population dynamics state‐process models together into a single SSM could allow estimation of bias parameters where the use of aggregated samples to construct indices do not. Accurate biological understanding through model fitting is likely to rely on the details of both state processes and observation error. Model information as defined here involved how deterministic drivers of population dynamics, process stochasticity, and observation noise, come together to affect inference about model parameters in particular. These are elements of a more general conceptual framework about system information and predictability (Pennekamp et al., 2019), the application of which could provide a better understanding of both parameter inference and system insights gained when fitting SSMs using biased data.

## AUTHOR CONTRIBUTIONS

**Leo Polansky:** Conceptualization (equal); data curation (equal); formal analysis (equal); methodology (equal); writing – original draft (equal); writing – review and editing (equal). **Lara Mitchell:** Conceptualization (equal); data curation (equal); formal analysis (equal); methodology (equal); writing – original draft (equal); writing – review and editing (equal). **Ken Newman:** Conceptualization (equal); data curation (equal); formal analysis (equal); methodology (equal); writing – original draft (equal); writing – review and editing (equal).

## CONFLICT OF INTEREST STATEMENT

All authors declare no conflicts of interest.

## Supporting information


Appendix S1

Appendix S2

Appendix S3

## Data Availability

Case study data is available at the Environmental Data Initiative portal: https://portal.edirepository.org/nis/mapbrowse?packageid=edi.1433.1 and https://portal.edirepository.org/nis/home.jsp.
