# Supplementary material for: Combining multiple data sources with different biases in state‐space models for population dynamics
Source: Ecol Evol. 2023 Jun 8;13(6):e10154. doi: 10.1002/ece3.10154 (PMC10249046; doi:10.1002/ece3.10154)
Supplement: Supplementary file 1 — Appendix S1 [file ECE3-13-e10154-s001.pdf]

Appendices

Combining multiple data sources with different biases in state-space models for population dynamics

Leo Polansky, Lara Mitchell, and Ken B. Newman

Appendix S1

Model identifiability

Section A Symbolic differentiation to detect parameter redundancy

This section evaluates model parameter redundancy (i.e. non-identifiability) based on the symbolic differentiation approach described by Catchpole & Morgan (1997) and extended to state-space models by Cole & McCrea (2016). Here we follow the notation of Cole (2020). For a model with parameter vector $\boldsymbol{\theta} = \{\theta_1, \ldots, \theta_q\}$ of length q, an exhaustive summary is the column vector $\boldsymbol{\kappa}$ of modeled observation expectations and variances,

$$\boldsymbol{\kappa} = \begin{bmatrix} E[y_{n,0}] \\ V[y_{n,0}] \\ E[y_{1,1}] \\ V[y_{1,1}] \\ E[y_{1,2}] \\ V[y_{1,2}] \\ \vdots \end{bmatrix} \tag{A.1}$$

where $y_{i,t}$ is the observed log-abundance for state i for cohort t. The derivative matrix is defined by

$$\mathbf{D} = \begin{bmatrix} \partial\kappa_1/\partial\theta_1 & \partial\kappa_2/\partial\theta_1 & \cdots & \partial\kappa_n/\partial\theta_1 \\ \partial\kappa_1/\partial\theta_2 & \partial\kappa_2/\partial\theta_2 & \cdots & \partial\kappa_n/\partial\theta_2 \\ \vdots & & & \vdots \\ \partial\kappa_1/\partial\theta_q & \partial\kappa_2/\partial\theta_q & \cdots & \partial\kappa_n/\partial\theta_q \end{bmatrix} \tag{A.2}$$

and the rank of $\mathbf{D}$ is computed. If the rank of $\mathbf{D}$ equals q then all parameters of $\boldsymbol{\theta}$ are in theory estimable. In particular, we will first study $\mathbf{D}_1(\boldsymbol{\theta}_1)$ derived from $\boldsymbol{\kappa}_1(\boldsymbol{\theta}_1)$ based on the deterministic version of the model where $\boldsymbol{\theta}_1$ contains all but the process variance or observation error variance parameters, and then extend this model to the case where these parameters are included. Let $\boldsymbol{\theta}_2$ represent these additional parameters, $\mathbf{D}_{2,2}(\boldsymbol{\theta}_2) = \partial\boldsymbol{\kappa}_2/\partial\boldsymbol{\theta}_2$ the corresponding derivative matrix, and $\boldsymbol{\theta}' = \{\boldsymbol{\theta}_1, \boldsymbol{\theta}_2\}$ the complete set of model parameters. Then the Extension Theorem (Section 3.2.6.1 of Cole [2020]) provides that if $\mathbf{D}_1(\boldsymbol{\theta}_1)$ and $\mathbf{D}_{2,2}(\boldsymbol{\theta}_2)$ are both of full rank, then the extended model will be full rank.

We describe the exhaustive summary and derivative matrix for a sequential life stage model with $n = 2$ and $n = 3$ stages where each vital rate is modeled as a function of a single covariate and observation error variances are stage-specific but constant in time. We use the resulting derivative matrces to identify general parameter redundancy features of these model types, and extend the results to cases in which n is arbitrary, covariates are removed from the vital rates, and observation error variances are time-specific. Because many of the terms are more simply expressed when using log-abundances, and for consistency with subsequent complete data likelihood descriptions (Section Section B) and model code formulations related to using a Laplace approximation, we work with log-abundances. Let $x_{i,t} = \log(N_{i,t})$ and $y_{i,t} = \log(\widehat{N}_{i,t})$ be the true and observed log-abundances for stage i of cohort t, respectively, and let $x_0 = \log(N_{2,0})$ be the initial stage two true log-abundance (x_0 is a parameter and hence the subscript labeling is slightly different from later-in-time

latent abundances). The model is

$$\text{Recruitment: } \log(\rho_t) = \beta_{R,0} + \beta_{R,1} w_{R,t} + \epsilon_{p,R,t},\ \epsilon_{R,t} \sim N(0, \sigma^2_{p,R}) \tag{A.3}$$

$$\begin{aligned}\text{Survival: } \log(\phi_{1,t}) =& \beta_{S_1,0} + \beta_{S1,1} w_{S_1,t} + \epsilon_{p,S_1,t} \\ & - \log(1 + \exp(\beta_{S_1,0} + \beta_{S1,1} w_{S_1,t} + \epsilon_{S_1,t})),\ \epsilon_{S_1,t} \sim N(0, \sigma^2_{p,S_1})\end{aligned} \tag{A.4}$$

$$\text{State process model: } x_{i,t} = \begin{cases} \log(\rho_t) + x_{2,t-1}, \text{ for } i = 1 \\ \log(\phi_{1,t}) + x_{1,t}, \text{ for } i = 2 \end{cases} \tag{A.5}$$

$$\text{Observation model: } y_{n,0} = \log(\psi_0) + x_{n,0} + \eta_{n,0},\ \eta_{n,0} \sim N(0, \sigma^2_{o,0}) \tag{A.6}$$

$$y_{i,t} = \log(\psi_i) + x_{i,t} + \eta_{i,t},\ \eta_{i,t} \sim N(0, \sigma^2_{o,i}) \tag{A.7}$$

and has parameter vector $\boldsymbol{\theta}' = \{\boldsymbol{\theta}_1, \boldsymbol{\theta}_2\}$ where $\boldsymbol{\theta}_1 = \{x_0, \psi_0, \psi_1, \psi_2, \beta_{R,0}, \beta_{R,1}, \sigma_{p,R}, \beta_{S_1,0}, \beta_{S_1,1}\}$ are the parameters related to the deterministic portions of the model and $\boldsymbol{\theta}_2 = \{\sigma_{p,R}, \sigma_{p,S_1}, \sigma_{o,0}, \sigma_{o,1}, \sigma_{o,2}\}$ are the process and observation error variance parameters. An alternative description of the latent state values $x_{i,t}$ that is helpful for describing the derivative matrix is

$$x_{i,t} = \begin{cases} x_0 + \log(\rho_1) + \log(\phi_{1,1}) + \cdots + \log(\rho_t), \text{ for } i = 1 \\ x_0 + \log(\rho_1) + \log(\phi_{1,1}) + \cdots + \log(\rho_t) + \log(\phi_{1,t}) + x_{1,t}, \text{ for } i = 2. \end{cases} \tag{A.8}$$

Denote

$$r_t = \beta_{R,0} + \beta_{R,1} w_{R,t} \tag{A.9}$$

$$s_{1,t} = \beta_{S_1,0} + \beta_{S_1,1} w_{S_1,1} - \log(1 + \exp(\beta_{S_1,0} + \beta_{S_1,1} w_{S_1,t})) \tag{A.10}$$

as the deterministic versions of the log-transformed vital rate models. The delta method based approximation to the variance of $\log(\phi_{1,t})$ is[1] $V[\log(\phi_{1,t})] \approx \sigma^2_{p,S_1}/(1 + \exp(\beta_{S_1,0} +$

[1]Dropping subscripts to the extent possible, $V[\log(\phi)] = V[f(\epsilon)]$ where $f(\epsilon) = \beta_0 + \beta_1 w + \epsilon - \log(1 +$

$\beta_{S_1,1} w_{S_1,t}))^2 = \sigma^2_{p,S_1} \tilde{s}^2_{1,t}$, where $\tilde{s}_{1,t} = 1/(1 + \exp(\beta_{S_1,0} + \beta_{S_1,1} w_{S_1,t}))$.

With this notation, first assume a model with no process and observation error variance parameters. Then

$$\boldsymbol{\kappa}_1(\boldsymbol{\theta}_1) = \begin{bmatrix} E[y_{2,0}] \\ E[y_{1,1}] \\ E[y_{2,1}] \\ E[y_{1,2}] \\ E[y_{2,2}] \\ \vdots \end{bmatrix} = \begin{bmatrix} \log(\psi_0) + x_0 \\ \log(\psi_1) + x_0 + r_1 \\ \log(\psi_2) + x_0 + r_1 + s_{1,1} \\ \log(\psi_1) + x_0 + r_1 + s_{1,1} + r_2 \\ \log(\psi_2) + x_0 + r_1 + s_{1,1} + r_2 + s_{1,2} \\ \vdots \end{bmatrix} \tag{A.11}$$

$\exp(\beta_0 + \beta_1 w + \epsilon))$. $f'(\epsilon) = 1 - \exp(\beta_0 + \beta_1 w + \epsilon)/(1 + \exp(\beta_0 + \beta_1 w + \epsilon)) = 1/(1 + \exp(\beta_0 + \beta_1 w + \epsilon))$. Then $V[\log(\phi)] \approx \sigma^2 f'(\epsilon)^2|_{\epsilon=0} = \sigma^2 \tilde{s}^2$, where $\tilde{s} = 1/(1+\exp(\beta_0+\beta_1 w)$ and we have expanded the function around $E[\epsilon] = 0$. Another way to see this is $V[\log(\phi)] = V[\log(\text{expit}(\text{logit}(\phi))] = V[\log(\text{expit}(A))] = V[\log(\exp(A)/(1 + \exp(A)))] = V[A - \log(1 + \exp(A))] = (1 - \exp(A)/(1 + \exp(A)))^2 V[A] = 1/(1 + \exp(A))^2 V[A] = 1/(1+\exp(A))^2 \sigma^2_{p,S_1}$ where the expansion is around the median value of ϕ, $A = \text{expit}(\phi)$, and expit is the inverse of the logit function.

where the relationship is exact because there is no randomness, and

$$
\mathbf{D}_1(\boldsymbol{\theta}_1) =
$$

$$
\begin{array}{c} \\ \partial/\partial x_0 \\ \partial/\partial \psi_0 \\ \partial/\partial \psi_1 \\ \partial/\partial \psi_2 \\ \partial/\partial \beta_{R,0} \\ \partial/\partial \beta_{R,1} \\ \partial/\partial \beta_{S_1,0} \\ \partial/\partial \beta_{S_1,1} \end{array}
\begin{array}{c} \begin{array}{cccccc} E[y_{2,0}] & E[y_{1,2}] & E[y_{2,1}] & E[y_{1,2}] & E[y_{2,2}] & \cdots \end{array} \\
\left[\begin{array}{cccccc}
1 & 1 & 1 & 1 & 1 & \cdots \\
1/\psi_0 & 0 & 0 & 0 & 0 & \cdots \\
0 & 1/\psi_1 & 0 & 1/\psi_1 & 0 & \cdots \\
0 & 0 & 1/\psi_2 & 0 & 1/\psi_2 & \cdots \\
0 & 1 & 1 & 2 & 2 & \cdots \\
0 & w_{R,1} & w_{R,1} & (w_{R,1}+w_{R,2}) & (w_{R,1}+w_{R,2}) & \cdots \\
0 & 0 & \tilde{s}_{1,1} & \tilde{s}_{1,1} & (\tilde{s}_{1,1}+\tilde{s}_{1,2}) & \cdots \\
0 & 0 & w_{S_1,1}\tilde{s}_{1,1} & w_{S_1,1}\tilde{s}_{1,1} & (w_{S_1,1}\tilde{s}_{1,1}+w_{S_1,2}\tilde{s}_{1,2}) & \cdots
\end{array}\right] \end{array}.
\tag{A.12}
$$

Extending the model to include variability in observations from both process variance and observation error,

$$
\boldsymbol{\kappa}_2(\boldsymbol{\theta}') = \begin{bmatrix} V[y_{2,0}] \\ V[y_{1,1}] \\ V[y_{2,1}] \\ V[y_{1,2}] \\ V[y_{2,2}] \\ \vdots \end{bmatrix} \approx \begin{bmatrix} \sigma^2_{o,0} \\ \sigma^2_{o,1} + \sigma^2_{p,R} \\ \sigma^2_{o,2} + \sigma^2_{p,R} + \sigma^2_{p,S_1}\tilde{s}^2_{1,1} \\ \sigma^2_{o,1} + 2\sigma^2_{p,R} + \sigma^2_{p,S_1}\tilde{s}^2_{1,1} \\ \sigma^2_{o,2} + 2\sigma^2_{p,R} + \sigma^2_{p,S_1}(\tilde{s}^2_{1,1} + \tilde{s}^2_{1,2}) \\ \vdots \end{bmatrix}
\tag{A.13}
$$

gives

$$\mathbf{D}_{2,2}(\boldsymbol{\theta}_2) =$$

$$\begin{array}{c} \\ \partial/\partial\sigma_{p,R} \\ \partial/\partial\sigma_{p,S_1} \\ \partial/\partial\sigma_{o,0} \\ \partial/\partial\sigma_{o,1} \\ \partial/\partial\sigma_{o,2} \end{array} \begin{array}{c} \begin{array}{cccccc} V[y_{2,0}] & V[y_{1,1}] & V[y_{2,1}] & V[y_{1,2}] & V[y_{2,2}] & \cdots \end{array} \\ \left[\begin{array}{cccccc} 0 & 2\sigma_{p,R} & 2\sigma_{p,R} & 4\sigma_{p,R} & 4\sigma_{p,R} & \cdots \\ 0 & 0 & 2\sigma_{p,S_1}\tilde{s}_{1,1}^2 & 2\sigma_{p,S_1}\tilde{s}_{1,1}^2 & 2\sigma_{p,S_1}(\tilde{s}_{1,1}^2 + \tilde{s}_{1,2}^2) & \cdots \\ 2\sigma_{o,0} & 0 & 0 & 0 & 0 & \cdots \\ 0 & 2\sigma_{o,1} & 0 & 2\sigma_{o,1} & 0 & \cdots \\ 0 & 0 & 2\sigma_{o,2} & 0 & 2\sigma_{o,2} & \cdots \end{array}\right] \end{array}. \quad \text{(A.14)}$$

When $n = 3$,

$$\mathbf{D}_1 =$$

$$\begin{array}{c} \\ \partial/\partial x_0 \\ \partial/\partial\psi_0 \\ \partial/\partial\psi_1 \\ \partial/\partial\psi_2 \\ \partial/\partial\psi_3 \\ \partial/\partial\beta_{R,0} \\ \partial/\partial\beta_{R,1} \\ \partial/\partial\beta_{S_1,0} \\ \partial/\partial\beta_{S_1,1} \\ \partial/\partial\beta_{S_2,0} \\ \partial/\partial\beta_{S_2,1} \end{array} \begin{array}{c} \begin{array}{ccccccccc} E[y_{3,0}] & E[y_{1,1}] & E[y_{2,1}] & E[y_{3,1}] & E[y_{1,2}] & E[y_{2,2}] & E[y_{3,2}] & E[y_{1,3}] & \cdots \end{array} \\ \left[\begin{array}{ccccccccc} 1 & 1 & 1 & 1 & 1 & 1 & 1 & 1 & \cdots \\ 1/\psi_0 & 0 & 0 & 0 & 0 & 0 & 0 & 0 & \cdots \\ 0 & 1/\psi_1 & 0 & 0 & 1/\psi_1 & 0 & 0 & 1/\psi_1 & \cdots \\ 0 & 0 & 1/\psi_2 & 0 & 0 & 1/\psi_2 & 0 & 0 & \cdots \\ 0 & 0 & 0 & 1/\psi_3 & 0 & 0 & 1/\psi_3 & 0 & \cdots \\ 0 & 1 & 1 & 1 & 2 & 2 & 2 & 3 & \cdots \\ 0 & w_{R,1} & w_{R,1} & w_{R,1} & (w_{R,1} + w_{R,2}) & (w_{R,1} + w_{R,2}) & (w_{R,1} + w_{R,2}) & (w_{R,1} + w_{R,2} + w_{R,3}) & \cdots \\ 0 & 0 & \tilde{s}_{1,1} & \tilde{s}_{1,1} & \tilde{s}_{1,1} & (\tilde{s}_{1,1} + \tilde{s}_{1,2}) & (\tilde{s}_{1,1} + \tilde{s}_{1,2}) & (\tilde{s}_{1,1} + \tilde{s}_{1,2}) & \cdots \\ 0 & 0 & w_{S_1,1}\tilde{s}_{1,1} & w_{S_1,1}\tilde{s}_{1,1} & w_{S_1,1}\tilde{s}_{1,1} & (w_{S_1,1}\tilde{s}_{1,1} + w_{S_1,2}\tilde{s}_{1,2}) & (w_{S_1,1}\tilde{s}_{1,1} + w_{S_1,2}\tilde{s}_{1,2}) & (w_{S_1,1}\tilde{s}_{1,1} + w_{S_1,2}\tilde{s}_{1,2}) & \cdots \\ 0 & 0 & 0 & \tilde{s}_{2,1} & \tilde{s}_{2,1} & \tilde{s}_{2,1} & (\tilde{s}_{2,1} + \tilde{s}_{2,2}) & (\tilde{s}_{2,1} + \tilde{s}_{2,2}) & \cdots \\ 0 & 0 & 0 & w_{S_2,1}\tilde{s}_{2,1} & w_{S_2,1}\tilde{s}_{2,1} & w_{S_2,1}\tilde{s}_{2,1} & (w_{S_2,1}\tilde{s}_{2,1} + w_{S_2,2}\tilde{s}_{2,2}) & (w_{S_2,1}\tilde{s}_{2,1} + w_{S_2,2}\tilde{s}_{2,2}) & \cdots \end{array}\right] \end{array} \quad \text{(A.15)}$$

and

$$
\mathbf{D}_{2,2}(\boldsymbol{\theta}_2) = \begin{array}{c} \\ \partial/\partial\sigma_{p,R} \\ \partial/\partial\sigma_{p,S_1} \\ \partial/\partial\sigma_{p,S_2} \\ \partial/\partial\sigma_{o,0} \\ \partial/\partial\sigma_{o,1} \\ \partial/\partial\sigma_{o,2} \\ \partial/\partial\sigma_{o,3} \end{array} \begin{array}{c} \begin{array}{cccccccc} V[y_{3,0}] & V[y_{1,1}] & V[y_{2,1}] & V[y_{3,1}] & V[y_{1,2}] & V[y_{2,2}] & V[y_{3,2}] & \cdots \end{array} \\ \left[\begin{array}{cccccccc} 0 & 2\sigma_{p,R} & 2\sigma_{p,R} & 2\sigma_{p,R} & 4\sigma_{p,R} & 4\sigma_{p,R} & 4\sigma_{p,R} & \cdots \\ 0 & 0 & 2\sigma_{p,S_1}\tilde{s}_{1,1}^2 & 2\sigma_{p,S_1}\tilde{s}_{1,1}^2 & 2\sigma_{p,S_1}\tilde{s}_{1,1}^2 & 2\sigma_{p,S_1}(\tilde{s}_{1,1}^2 + \tilde{s}_{1,2}^2) & 2\sigma_{p,S_1}(\tilde{s}_{1,1}^2 + \tilde{s}_{1,2}^2) & \cdots \\ 0 & 0 & 0 & 2\sigma_{p,S_2}\tilde{s}_{2,1}^2 & 2\sigma_{p,S_2}\tilde{s}_{2,1}^2 & 2\sigma_{p,S_2}\tilde{s}_{2,1}^2 & 2\sigma_{p,S_2}(\tilde{s}_{2,1}^2 + \tilde{s}_{2,2}^2) & \cdots \\ 2\sigma_{o,0} & 0 & 0 & 0 & 0 & 0 & 0 & \cdots \\ 0 & 2\sigma_{o,1} & 0 & 0 & 2\sigma_{o,1} & 0 & 0 & \cdots \\ 0 & 0 & 2\sigma_{o,2} & 0 & 0 & 2\sigma_{o,2} & 0 & \cdots \\ 0 & 0 & 0 & 2\sigma_{o,3} & 0 & 0 & 2\sigma_{o,3} & \cdots \end{array}\right] \end{array}. \quad \text{(A.16)}
$$

With the understanding that fixing a parameter means removing the corresponding row from $\mathbf{D}$, and using Gaussian elimination arguments and notation where $aR1 + bR2 = R3$ means $a *$ row $1 + b *$ row $2 =$ row 3, some observations are about the $\mathbf{D}_1$ matrices are:

- Rows related to the x_0 and the ψ_i parameters are linearly dependent. For example, $\psi_0 R2 + \psi_1 R3 + \psi_2 R4 = R1$ of equation A.12 and $\psi_0 R2 + \psi_1 R3 + \psi_2 R4 + \psi_3 R5 = R1$ of equation A.15. Fixing any one of these rows removes this dependence.

- For a model with one bias parameter fixed and no survival vital rates covariates, rows related to the remaining bias and the vital rate intercept parameters will be linearly dependent. For example, fix ψ_0 in equation A.12 to remove redundancy between the ψ_1 and x_0. Without covariates, $\tilde{s}_{1,t_1} = \tilde{s}_{1,t_2}$ for any t_1 and t_2, and $1/\psi_1 R5 - 1/(\psi_1 \tilde{s}_{1,1}) R7 = R3$. Similarly, in equation A.15, $1/\psi_1 R6 - 1/(\psi_1 \tilde{s}_{1,1}) R8 = R3$ and $1/(\psi_2 \tilde{s}_{1,1}) R8 - 1/(\psi_2 \tilde{s}_{2,1}) R10 = R4$.

- Similarly, if two adjacent survival sub-models both do not have covariates, say stages i and $i+1$, rows corresponding to the partials with respect to intercept parameters $\beta_{S_i,0}$ and $\beta_{S_{i+1},0}$, and ψ_{i+1} are dependent. For example, in equation A.15 $1/(\psi_2 \tilde{s}_{1,1}) R8 - 1/(\psi_2 \tilde{s}_{2,1}) R10 = R4$.

- For each vital rate sub-model, at least two observations with different covariate values are needed for the rows to be linearly independent. In equation A.15, for example, if

$w_{R,1} = w_{R,2} = w_{R,3}$, then $1/w_{R,1} R7 = R6$.

- The rows for the parameters between different vital rates are linearly independent (leading zeros in columns for later stage vital rates cannot cancel earlier non-zero entries for earlier stage vital rate parameters).

Some observations are about the $\mathbf{D}_{2,2}$ matrices are:

- Without covariates, there is linear dependence between rows related to some of the observation error variance parameters and the vital rate process variance parameters. For example, in equation A.14, without covariates $\tilde{s}^2_{1,t}$ is constant for all t and $\sigma_{p,R}/\sigma_{o,1} R3 + \sigma_{p,R}/(\sigma_{p,S_1} \tilde{s}^2_{1,1}) R2 = R1$. Similarly, in equation A.16, without covariates $\sigma_{p,R}/\sigma_{o,1} R4 + \sigma_{p,R}/(\sigma_{p,S_1} \tilde{s}^2_{1,1}) R2 = R1$.
- Removing the observation error variance parameters, and the resulting first column of all zeros, results in a matrix that has full rank irrespective of covariates.

Taken together, these observations based on $n = 2$ and $n = 3$ imply the derivative matrix $\mathbf{D}_1$ with one ψ_i parameter fixed and vital rates that depend on covariates has full rank. With covariates the $\mathbf{D}_{2,2}$ matrices also have full rank. By the Extension Theorem, a model including bias, covariates, process variance, and observation error variances is estimable.

Assume a model with n stages is estimable, and extend this model to have $n + 1$ stages where, without loss of generality, the added stage comes between stage n and stage 1 of the n-stage model. Then

$$\mathbf{D}_{2,2}(\psi_{n+1}, \beta_{S_{n+1},0}, \beta_{S_{n+1},1}, \sigma_{p,S_{n+1}}, \sigma_{o,n+1}) =$$

$$\begin{array}{c} \\ \partial/\partial\psi_{n+1} \\ \partial/\partial\beta_{S_{n+1},0} \\ \partial/\partial\beta_{S_{n+1},1} \\ \partial/\partial\sigma_{p,S_{n+1}} \\ \partial/\partial\sigma_{o,n+1} \end{array} \begin{array}{c} \begin{array}{ccccc} E[y_{n+1,1}] & V[y_{n+1,2}] & E[y_{n+1,2}] & V[y_{n+1,2}] & \cdots \end{array} \\ \left[\begin{array}{ccccc} 1/\psi_{n+1} & 0 & 1/\psi_{n+1} & 0 & \cdots \\ \tilde{s}_{n+1,1} & 0 & (\tilde{s}_{n+1,1} + \tilde{s}_{n+1,2}) & 0 & \cdots \\ w_{S_{n+1},1}\tilde{s}_{n+1,1} & 0 & (w_{S_{n+1},1}\tilde{s}_{n+1,1} + w_{S_{n+1},2}\tilde{s}_{n+1,2}) & 0 & \cdots \\ 0 & 2\sigma_{p,S_{n+1}}\tilde{s}^2_{n+1,1} & 0 & 2\sigma_{p,S_{n+1}}(\tilde{s}^2_{n+1,1} + \tilde{s}^2_{n+1,2}) & \cdots \\ 0 & 2\sigma_{o,n+1} & 0 & 2\sigma_{o,n+1} & \cdots \end{array}\right] \end{array}. \tag{A.17}$$

Equation A.17 has full rank if there is a covariate, and by the Extension Theorem the extended model has full rank.

Section B Implications of fixing a reference observation bias parameter

The symbolic differentiation approach described in Section A indicates that at least initial (log) abundance x_0 or one of the bias parameters ψ_i must be fixed to avoid parameter redundancy. This section examines the implications of fixing one of these parameters based on an examination of the complete data likelihood (i.e. the joint probability distribution for the state process variables and the observations). Our objectives are to 1) show that the choice of which of these parameters is fixed and the value that it is fixed at does not impact inference about state process parameters, and 2) establish a relationship between the estimates of the x_0 and ψ_i parameters based on this choice. Here we allow for an arbitrary number of stages, n, assume observation variance parameters are time specific but known prior to model fitting, and allow more than one covariate per vital rate model, i.e.

$$\text{Recruitment } : \rho_t \sim \text{Log-Normal}\left(\mathbf{w}_{R,t}\boldsymbol{\beta}_R, \sigma^2_{P,R}\right) \tag{B.1}$$

$$\text{Survival } : \phi_{i,t} \sim \text{Logit-Normal}\left(\mathbf{w}_{i,t}\boldsymbol{\beta}_{S_i}, \sigma^2_{P,S_i}\right) \tag{B.2}$$

where $\boldsymbol{\beta}_R^T = (\beta_{R,0}, ..., \beta_{R,m_R})$ is a vector of $m_R + 1$ regression coefficients corresponding to a vector of recruitment predictor variables $x_{\cdot,t}$ and design matrix $\mathbf{w}_{R,t} = (1, x_{1,t}, ..., x_{m_R,t})$, and $\sigma^2_{p,R}$ is the recruitment process model variance on the log scale. The terms in the survival function equation B.2 are defined analogously but with possibly different dimensions reflecting stage-specific numbers of covariates used in survival predictions.

The fixed effect parameters are partitioned in $\boldsymbol{\theta} = \{\boldsymbol{\theta}_1, \boldsymbol{\theta}_2\}$ where

$$\boldsymbol{\theta}_1 = \{\boldsymbol{\beta}_R^T, \sigma_{p,R}, \boldsymbol{\beta}_{S_1}^T, \sigma_{p,S_1}, \ldots, \boldsymbol{\beta}_{S_n}^T, \sigma_{p,S_n}\}$$

are the state process model parameters and $\boldsymbol{\theta}_2 = \{x_0, \psi_1, \ldots, \psi_n\}$ are the initial abundance and bias parameters. Let u denote the sequence of latent random effects, $u = \{\rho_1, \phi_{1,1}, \phi_{2,1}, \ldots\}$, which in turn defines a set of latent abundances $X = \{x_0, x_0 + \log(\rho_1), x_0 + \log(\rho_1) + \log(\phi_{1,1}), \ldots\} = \{x_0, x_{1,1}, x_{2,1}, \ldots\}$ given an initial (log) abundance x_0, and let $Y = \{y_{n,0}, y_{1,1}, y_{2,1}, \ldots\}$ be the complete set of log transformed abundance observations. The joint likelihood (complete data likelihood) is

$$L(\boldsymbol{\theta}|Y, X) = L(\boldsymbol{\theta}|Y, u) = \underbrace{P(y_{n,0}|x_{n,0}) \prod_{i=1,t=1} P(y_{i,t}|x_{i,t})}_{\text{observation likelihood}} \underbrace{\prod_{i\geq 2,t\geq 1} P(x_{i,t}|x_{i-1,t})}_{\text{survival likelihood}} \underbrace{\prod_{t\geq 1} P(x_{1,t}|x_{n,t-1})}_{\text{recruitment likelihood}} \tag{B.3}$$

where the products are over all possible values of the indices given the specified lower bounds. The factors in equation B.3, written anticipating the use of the Laplace approximation to integrate out u, which benefits from random effects parameterized as normal random variables (Fournier *et al.*, 2012), are

$$\text{Recruitment model likelihood} : \quad P(x_{1,t}|x_{n,t-1}) = f(x_{1,t}; x_{n,t-1} + \mathbf{w}_{R,t}\boldsymbol{\beta}_R, \sigma^2_{P,R}) \tag{B.4}$$

$$\text{Survival model likelihood} : \quad P(x_{i,t}|x_{i-1,t}) = f(\text{logit}(\exp(x_{i,t} - x_{i-1,t})); \mathbf{w}_{i-1,t}\boldsymbol{\beta}_{S_i-1}, \sigma^2_{P,S_i-1}) \tag{B.5}$$

$$\text{Observation model likelihood} : \quad P(y_{i,t}|x_{i,t}) = f(y_{i,t}; \log(\psi_i) + x_{i,t}, \sigma^2_{o,i,t}) \tag{B.6}$$

where $f(x; \mu, \sigma^2)$ denotes the normal probability density function. The marginal likelihood that is optimized is $L(\theta|Y) = \int_u L(\theta|Y, u)du$. Note $\exp(x_{i,t} - x_{i-1,t})$ in Equation B.5 equals $\phi_{i-1,t}$.

Because each latent abundance $x_{i,t}$ can be written as $x_{i,t} = x_{n,0} + \sum_{j=1}^{n(t-1)+i} \log(u_j)$, the

parameters $x_{n,0} = \log(N_{n,0})$ and ψ_i only occur in pairs within the factor B.6 of equation B.3, and are completely confounded. Given $\boldsymbol{\theta}$ and an arbitrary constant $a > 0$ define $\boldsymbol{\theta}'$ by replacing $N_{n,0}$ and ψ_i in $\boldsymbol{\theta}$ with $N'_{n,0} = aN_{n,0}$ (equivalently $x'_{n,0} = x_{n,0} + \log(a)$) and $\psi'_i = \psi_i/a$ (equivalently $\log(\psi'_i) = \log(\psi_i) - \log(a)$). Then the μ term of Equation B.6 is

$$\begin{aligned}\mu &= \log(\psi_i) + x_{i,t} \\ &= \log(\psi_i) - \log(a) + x_{n,0} + \log(a) + \sum_{j=1}^{n(t-1)+i} \log(u_j) \\ &= \log(\psi'_i) + x'_{n,0} + \sum_{j=1}^{n(t-1)+i} \log(u_j)\end{aligned}$$

and $L(\boldsymbol{\theta}|Y) = L(\boldsymbol{\theta}'|Y)$.

Another observation based on $x_{i,t} = x_{n,0} + \sum_{j=1}^{n(t-1)+i} \log(u_j)$ is that the process likelihoods in Equation B.4 and Equation B.5 do not depend on the value of $x_{n,0}$, and hence do not depend on $\boldsymbol{\theta}_2$.

Select stage j to be the reference stage and fix its bias parameter value at the constant ψ_{ref-j}. Denote maximum likelihood estimates given ψ_{ref-j} as $\widehat{N}_{n,0|\psi_{ref-j}}$ and $\widehat{\psi}_{i\neq j|\psi_{ref-j}}$, where we have made explicit the dependence of these estimates on the reference choice ψ_{ref-j}. Choosing a new reference value $\psi'_{ref-j} = \psi_{ref-j}/a$, where $a > 0$, will result in maximum likelihood estimates $\widehat{N}'_{n,0|\psi'_{ref-j}} = a\widehat{N}_{n,0|\psi_{ref-j}}$ and $\widehat{\psi}'_{i|\psi'_{ref-j}} = \widehat{\psi}_{i|\psi_{ref-j}}/a$, but will otherwise not change the overall likelihood as shown above.

References

Catchpole, E.A. & Morgan, B.J.T. (1997) Detecting parameter redundancy. *Biometrika*, **84**, 187–196.

Cole, D. (2020) *Parameter Redundancy and Identifiability.* CRC Press, Taylor & Francis

Group, Boca Raton, Florida.

Cole, D.J. & McCrea, R.S. (2016) Parameter redundancy in discrete state-space and integrated models. *Biometrical Journal*, **58**, 1071–1090.

Fournier, D.A., Skaug, H.J., Ancheta, J., Ianelli, J., Magnusson, A., Maunder, M.N., Nielsen, A. & Sibert, J. (2012) Ad model builder: using automatic differentiation for statistical inference of highly parameterized complex nonlinear models. *Optimization Methods and Software*, **27**, 233–249.
