# Supplementary material for: Combining multiple data sources with different biases in state‐space models for population dynamics
Source: Ecol Evol. 2023 Jun 8;13(6):e10154. doi: 10.1002/ece3.10154 (PMC10249046; doi:10.1002/ece3.10154)
Supplement: Supplementary file 2 — Appendix S2 [file ECE3-13-e10154-s002.pdf]

Appendices

Combining multiple data sources with different biases in state-space models for population dynamics

Leo Polansky, Lara Mitchell, and Ken B. Newman

Appendix S2

Additional simulation experiment material

The full factorial simulation experiment was designed to study parameter inference across four scenarios based on whether or not abundance estimates are biased and whether or not bias parameters to be estimated are included in the observation model fit to the data. The fitted models were frequentist state-space models with Laplace approximation fit using `TMB`. Data was simulated using parameters shown in Table 1, and is distinguished by whether all abundance estimates are not biased ($\psi_i = 1$ for all i) or they are ($\psi_i \neq 1$ for each i). Fitted models were distinguished by whether they assumed abundance estimates were not biased ($\psi_i = 1$ for all i) or did, in which case the models included the maximum number of estimable bias parameters by setting $\psi_{4,\text{set}} = 1$ and estimating the remaining bias parameters. For each scenario 1,000 datasets were simulated and the two model types were fit to each simulation. A dataset and associated model parameter estimates was retained only if both model types successfully converged.

Table S2.1: Parameter estimate mean, standard deviation (SD), relative bias as a percent (Rel.Bias, 100*(mean-true)/true), and root mean square error (RMSE) for two different types of models when abundance indices are unbiased or biased. When observations are biased parameter estimate statistics use adjusted true values, $\psi_i * \psi_{ref-j}/\psi_j = 2\psi_i$ (the reference bias parameter is $\psi_{4,ref} = 1$ but the true value is $\psi_4 = 0.5$, see Section 4.1). 5% of the simulations associated with outlier estimates were excluded.

		Mean	SD	Rel. Bias	RMSE	Mean	SD	Rel. Bias	RMSE
		Unbiased observations							
Parameter	True	Model correctly assumes all $\psi_i = 1$				Model correctly assumes only $\psi_4 = 1$			
$\log(N_{n,0})$	13.82	13.82	0.09	-0.00	0.09	13.82	0.09	-0.00	0.09
$\beta_{R,0}$	1.00	1.00	0.10	0.35	0.10	1.09	0.24	9.25	0.26
$\beta_{R,1}$	-0.50	-0.50	0.10	-0.46	0.10	-0.50	0.10	-0.47	0.10
$\beta_{S_1,0}$	1.00	1.03	0.19	2.64	0.19	1.05	0.55	4.89	0.55
$\beta_{S_1,1}$	1.30	1.33	0.20	2.07	0.20	1.37	0.37	5.34	0.38
$\beta_{S_2,0}$	1.00	1.00	0.19	0.03	0.19	0.99	0.54	-0.74	0.54
$\beta_{S_2,1}$	1.30	1.30	0.20	-0.18	0.20	1.33	0.37	2.26	0.37
$\beta_{S_3,0}$	1.00	1.01	0.20	1.45	0.20	1.02	0.56	1.97	0.56
$\beta_{S_3,1}$	1.30	1.31	0.21	1.04	0.21	1.35	0.37	3.65	0.37
$\sigma_{p,R}$	0.50	0.46	0.07	-8.16	0.08	0.46	0.07	-8.34	0.08
σ_{p,S_1}	0.50	0.43	0.13	-14.55	0.15	0.41	0.15	-18.75	0.18
σ_{p,S_2}	0.50	0.43	0.12	-13.41	0.14	0.41	0.14	-18.27	0.17
σ_{p,S_3}	0.50	0.43	0.13	-14.66	0.15	0.41	0.15	-18.87	0.18
ψ_1	1.00					0.94	0.19	-6.14	0.20
ψ_2	1.00					0.96	0.16	-4.32	0.17
ψ_3	1.00					0.98	0.11	-1.68	0.11
ψ_4	1.00						Reference survey		
		Biased observations							
		Model incorrectly assumes all $\psi_i = 1$				Model only incorrectly assumes $\psi_4 = 1$			
$\log(N_{n,0})$	13.12	13.12	0.09	-0.05	0.09	13.12	0.09	0.00	0.09
$\beta_{R,0}$	1.00	0.31	0.10	-69.42	0.70	1.11	0.28	10.81	0.30
$\beta_{R,1}$	-0.50	-0.50	0.11	0.39	0.11	-0.50	0.10	-0.23	0.10
$\beta_{S_1,0}$	1.00	0.62	0.18	-38.09	0.42	1.02	0.55	1.90	0.55
$\beta_{S_1,1}$	1.30	1.05	0.25	-19.10	0.35	1.35	0.37	3.59	0.37
$\beta_{S_2,0}$	1.00	25.01	16.00	2400.79	28.84	1.00	0.61	-0.40	0.61
$\beta_{S_2,1}$	1.30	11.42	11.23	778.71	15.12	1.33	0.40	2.60	0.40
$\beta_{S_3,0}$	1.00	8.21	2.84	720.70	7.75	1.02	0.56	1.84	0.56
$\beta_{S_3,1}$	1.30	5.13	1.97	294.82	4.31	1.35	0.36	3.93	0.37
$\sigma_{p,R}$	0.50	0.50	0.08	0.43	0.08	0.46	0.07	-8.33	0.08
σ_{p,S_1}	0.50	0.85	0.17	70.05	0.39	0.41	0.15	-17.61	0.17
σ_{p,S_2}	0.50	11.53	23.11	2205.47	25.60	0.38	0.17	-23.87	0.21
σ_{p,S_3}	0.50	1.47	0.95	194.88	1.36	0.40	0.15	-19.37	0.18
ψ_1	0.20					0.37	0.08	-7.02	0.09
ψ_2	0.10					0.19	0.04	-4.62	0.04
ψ_3	0.40					0.79	0.09	-1.62	0.09
ψ_4	0.50						Reference survey		

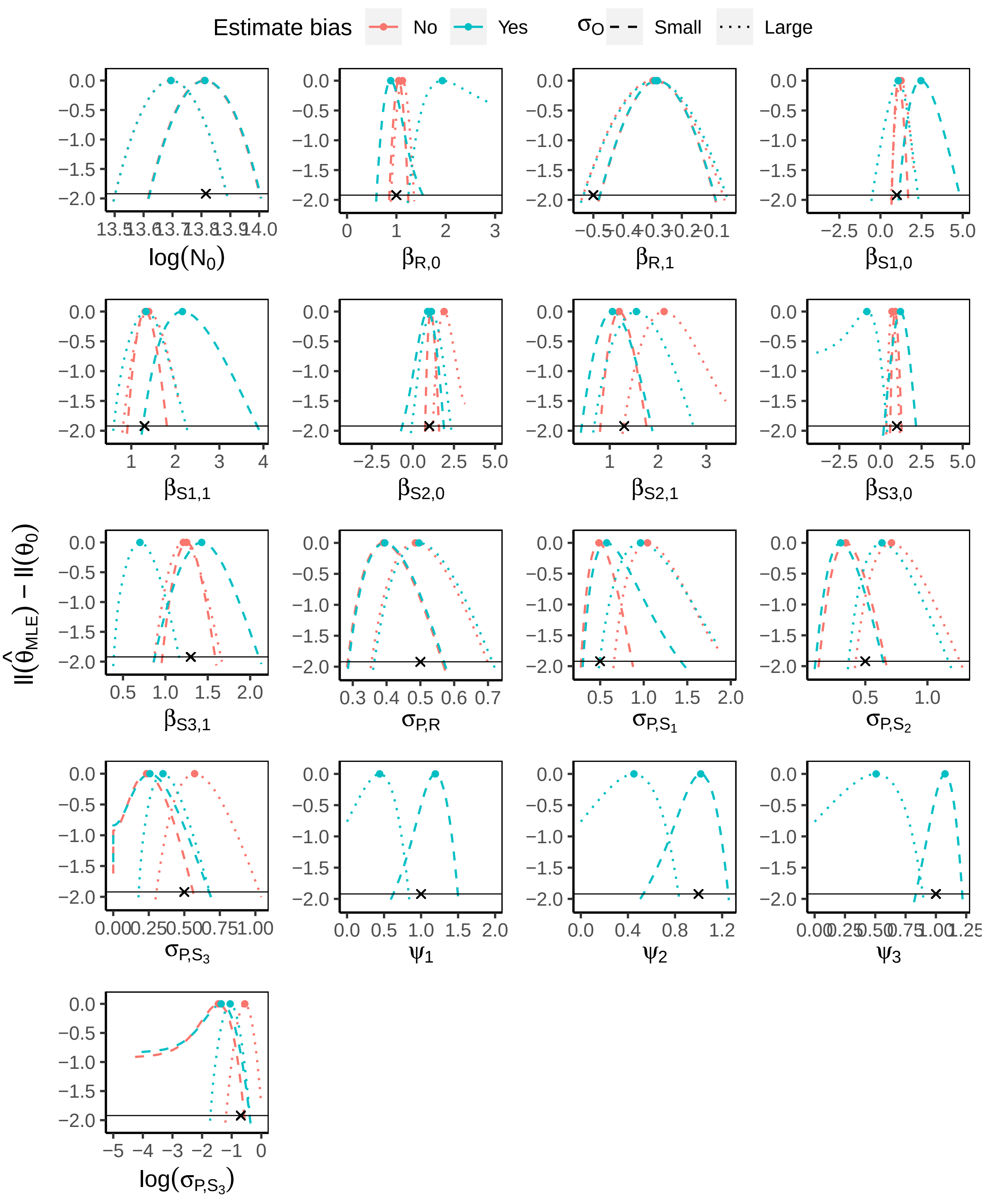

Figure S2.1: Profile log-likelihoods for parameters of models fit to simulated datasets generating unbiased abundance observations. True parameter values are at the x marks. Horizontal lines are drawn at the likelihood ratio test statistic for the 95% confidence interval. The observations here were generated using the same set of latent true abundance values, so differences are entirely attributable to observation error variance differences, being either small ($\sigma_o = 0.1$) or large ($\sigma_o = 0.5$).

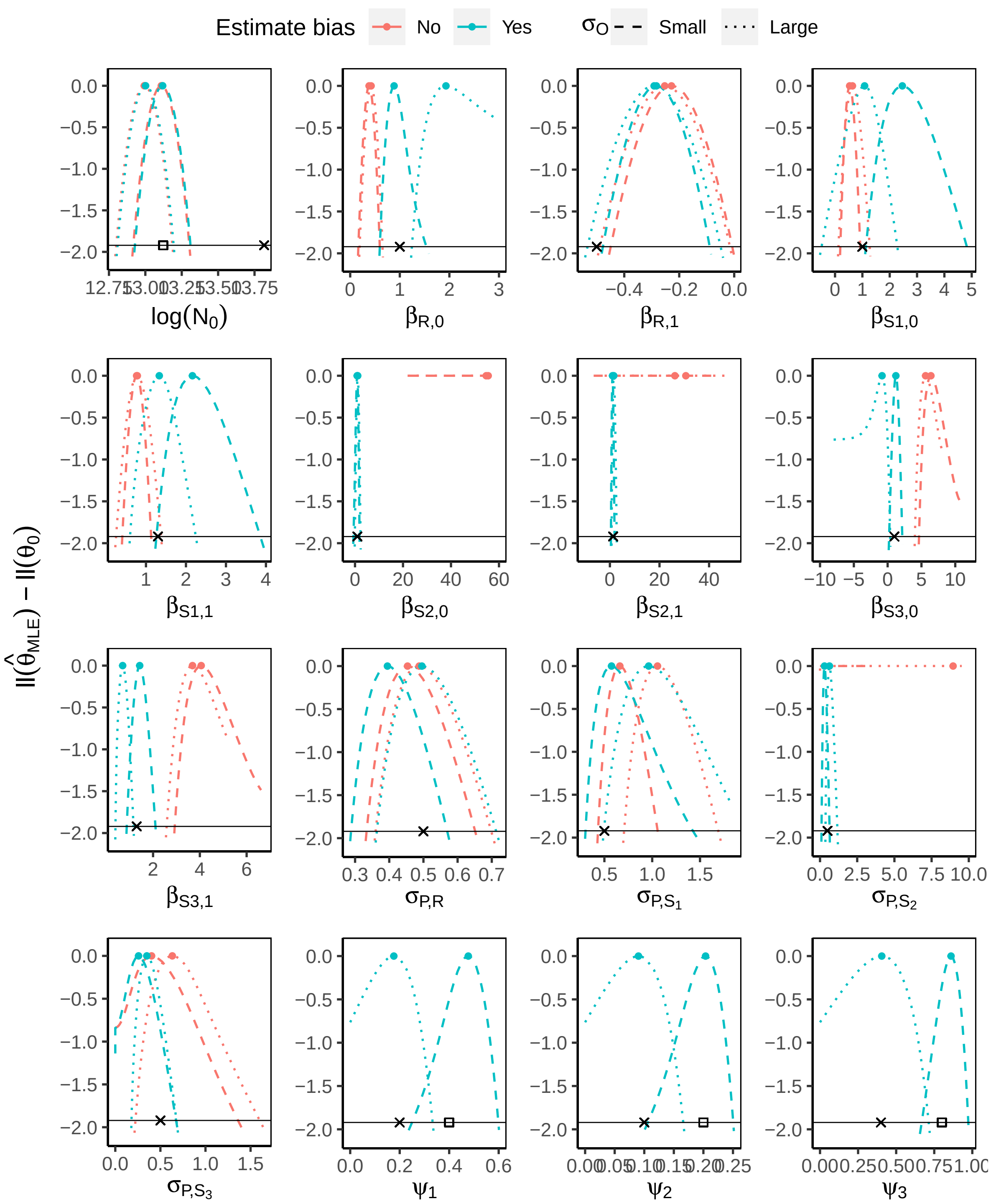

Figure S2.2: Profile log-likelihoods for parameters of models fit to simulated datasets generating biased abundance observations. Points along the axes are the true values (x's) and the adjusted target value (squares) when relevant, i.e. at $\log(N_{n,0}\psi_4/\psi_{4,\text{set}}) = \log(1e6/2)$ and $\psi_i\psi_{4,\text{set}}/\psi_4$ for $i = 1, 2, 3$. Horizontal lines are drawn at the likelihood ratio test statistic for the 95% confidence interval. The observations here were generated using the same set of latent true abundance values, so differences are entirely attributable to observation error variance differences, being either small ($\sigma_o = 0.1$) or large ($\sigma_o = 0.5$).

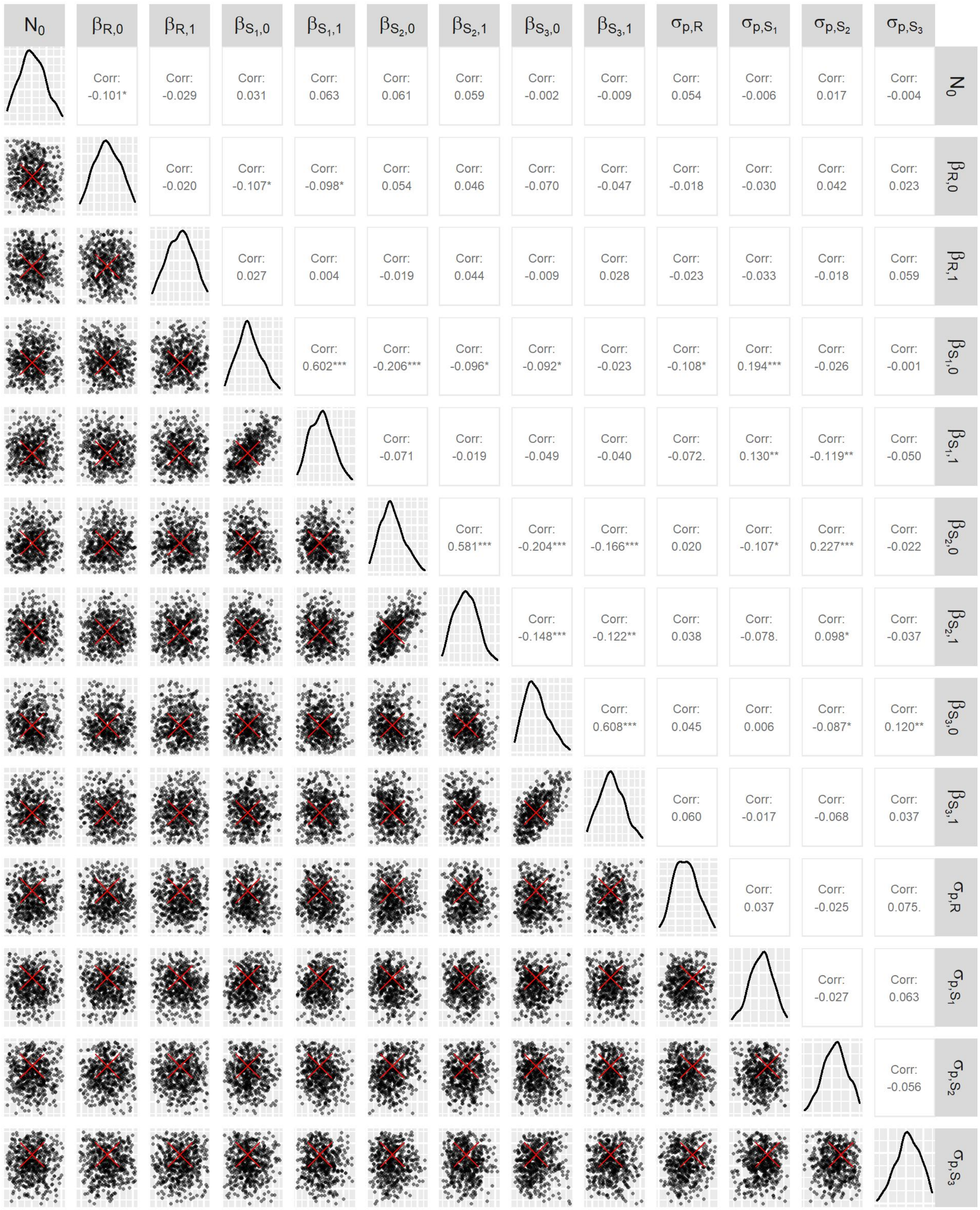

Figure S2.3: Pairwise maximum likelihood estimates (lower panels), estimate density (diagonal panels), and correlation (upper panels), when the data is not biased and the model does not estimate bias. Estimates outside the central 95% quantile interval are excluded for clarity. Red X marks are at the true parameter locations.

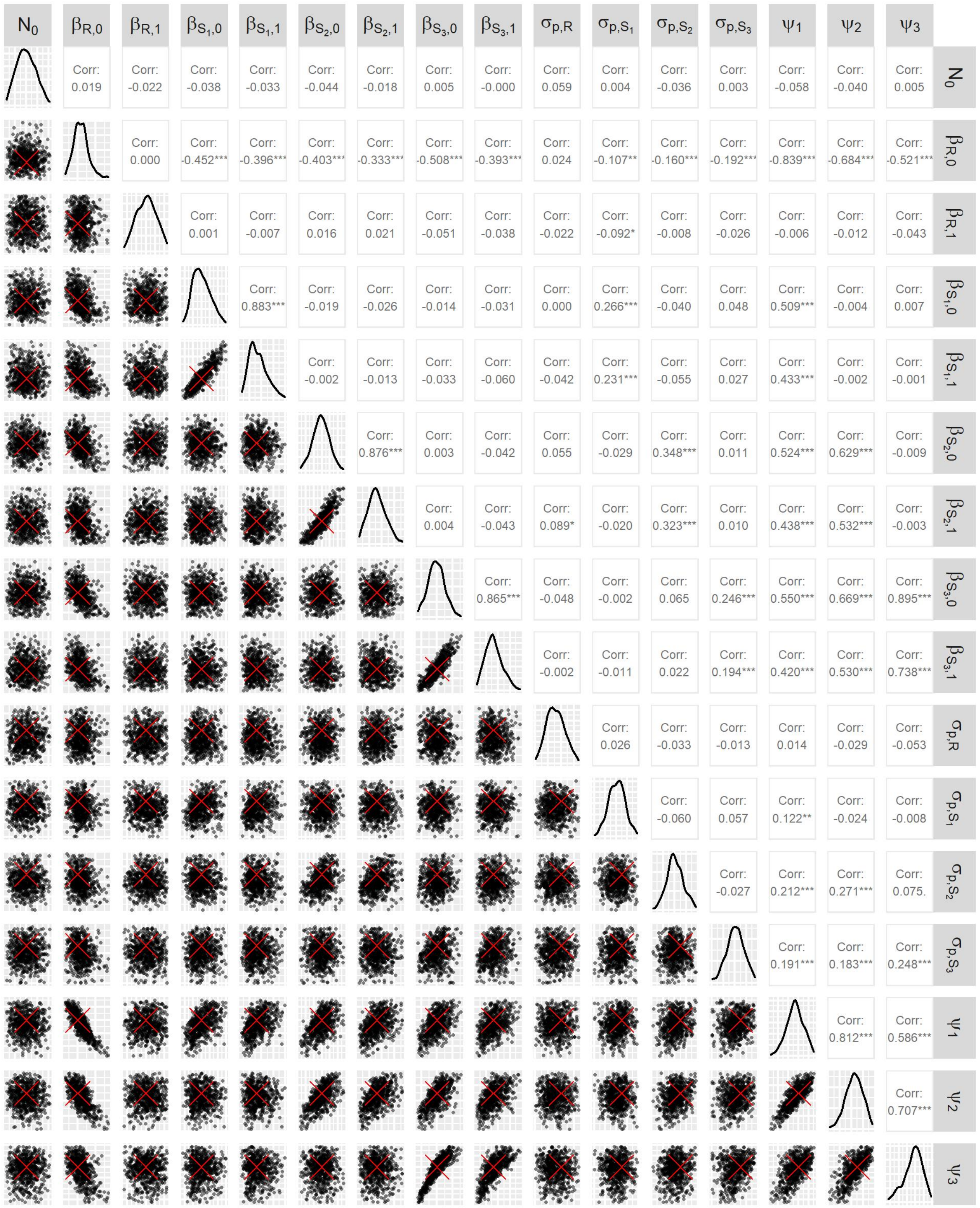

Figure S2.4: Pairwise maximum likelihood estimates (lower panels), estimate density (diagonal panels), and correlations (upper panels) when the data is not biased but the model estimates bias. Estimates outside the central 95% quantile interval are excluded for clarity. Red X marks are at the true values or adjusted target values.

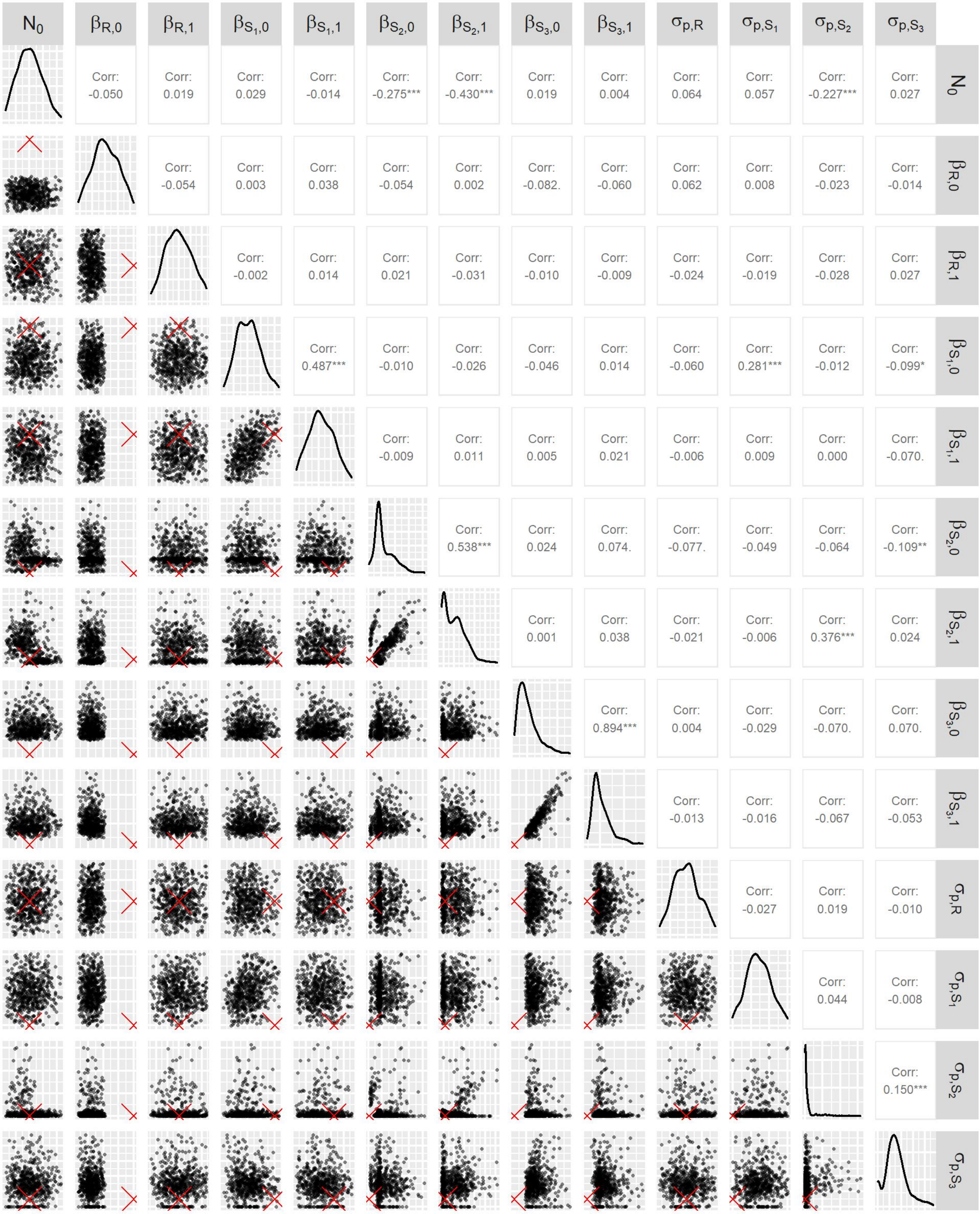

Figure S2.5: Pairwise maximum likelihood estimates (lower panels), estimate density (diagonal panels), and correlations (upper panels) when the data is biased but the model does not estimate bias. Estimates outside the central 95% quantile interval are excluded for clarity. Panel labeling as in Figure S2.3.

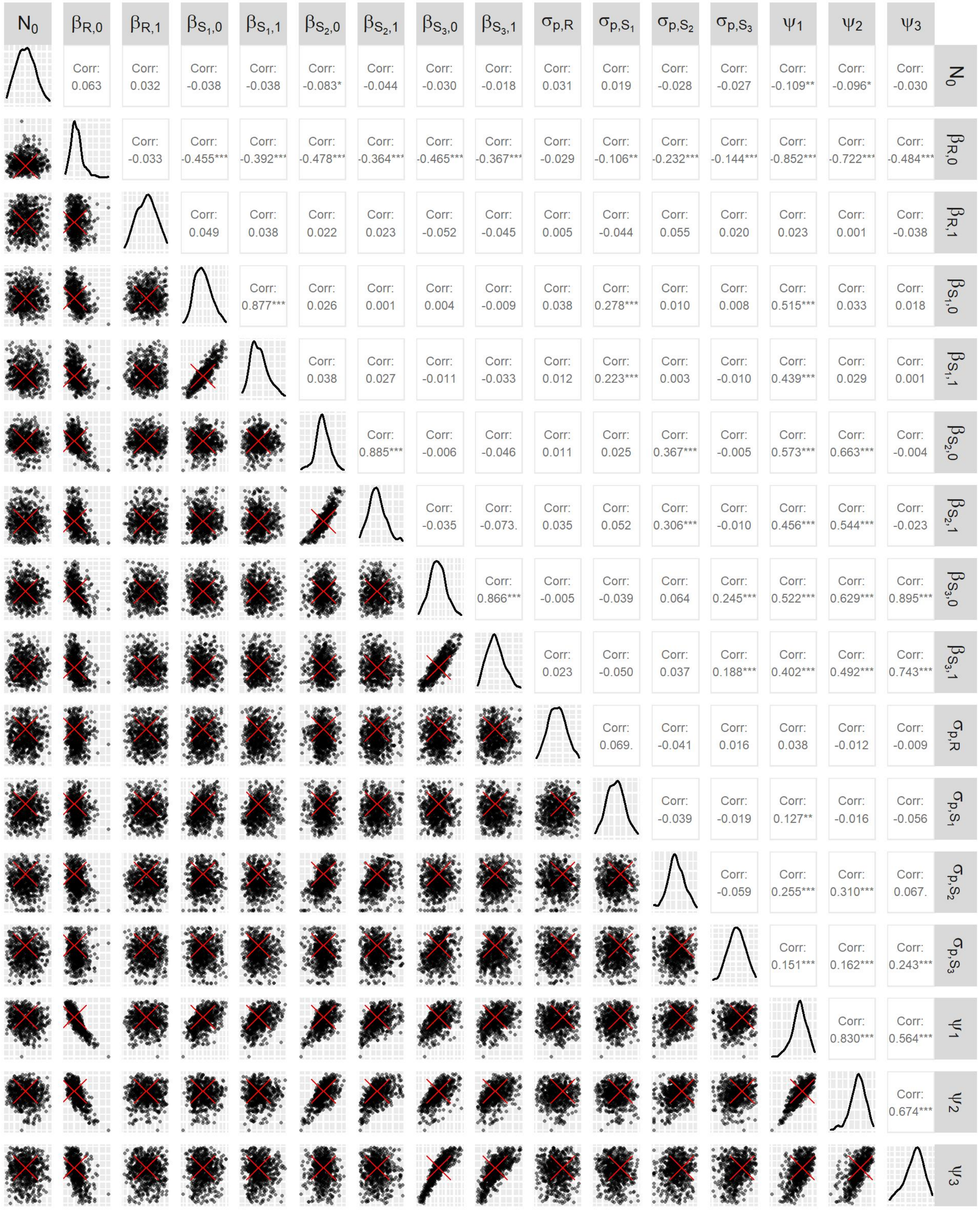

Figure S2.6: Pairwise maximum likelihood estimates (lower panels), estimate density (diagonal panels), and correlations (upper panels) when the data is biased and the model estimates bias. Estimates outside the central 95% quantile interval are excluded for clarity. Panel labeling as in Figure S2.5.
