# Supplementary material for: Combining multiple data sources with different biases in state‐space models for population dynamics
Source: Ecol Evol. 2023 Jun 8;13(6):e10154. doi: 10.1002/ece3.10154 (PMC10249046; doi:10.1002/ece3.10154)
Supplement: Supplementary file 3 — Appendix S3 [file ECE3-13-e10154-s003.pdf]

Appendices

Combining multiple data sources with different biases in state-space models for population dynamics

Leo Polansky, Lara Mitchell, and Ken B. Newman

Appendix S3

Additional delta smelt case study material

Table S3.1: Indices of potential biotic and abiotic conditions used as predictor variables for the delta smelt case study. Generally indices are means of monthly means of daily measurements collected during Mar-May, Jun-Aug, Sep-Nov, and Dec-Feb for ρ, ϕ_1, ϕ_2, and ϕ_3, respectively. The direction column shows the expected effect.

Predictor	Vital rates	Direction	Remarks
FebAdultLength	ρ	+	Average fork length (mm) of adult fish. Larger female fish are expected to produce more eggs.
ACM	ρ, ϕ_2, ϕ_3	+	Large prey availability for juveniles and adults.
NJ	ρ	+	Small prey availability for early life history fish.
OMR	ϕ_3	+	Old and Middle river flow index.
Outflow	ρ, ϕ_1	+	Outflow, an omnibus habitat condition measure indexing the amount of water moving towards the ocean.
SBAge1Plus	ϕ_3	−	Adult striped bass (*Morone saxatilis*), a predator.
Secchi	ρ, ϕ_1, ϕ_2	−	Water clarity measured by Secchi depth (cm).
South Secchi	ϕ_3	−	A water clarity index using Secchi data collected only from the south region of the Delta.
Temperature	ρ, ϕ_1	−	Temperature (deg C). Cooler temperatures are expected to be better for recruitment and summer survival.
LSZ	ϕ_2	+	Low salinity zone habitat volume. Increases during fall are expected to result in increased survival.
X2	ρ_t	−	Approximate location of the 2-ppt isohaline. $X2_{t-1}$ denotes the fall X2 value of the prior calendar year used in the recruitment model ρ_t.

Table S3.2: Delta smelt log abundances log$N_{\cdot,t}$ and observation standard errors $\sigma_{o,\cdot,t}$. The initial observation is related to the 1990 cohort stage 4 abundance. The delta smelt life cycle model is such that the first three stages have birth cohort and calendar years that match, but the stage four cohort corresponds to calendar year + 1. Stage four abundances were estimated using two surveys, the first (MWT) was used to estimate the 1990-2000 cohorts and the second (SKT) was used to estimate the 2001 through 2015 cohorts.

Cohort	$\log(N_{s_1,t})$	$\log(N_{s_2,t})$	$\log(N_{s_3,t})$	$\log(N_{s_4,t})$	$\sigma_{o,1,t}$	$\sigma_{o,2,t}$	$\sigma_{o,3,t}$	$\sigma_{o,4,t}$
1990				11.78				0.25
1991		15.19	13.98	11.55		0.17	0.19	0.25
1992		14.36	11.96	10.93		0.17	0.36	0.25
1993		15.89	14.44	13.09		0.18	0.29	0.22
1994		15.54	11.04	11.41		0.13	0.38	0.26
1995	15.15	15.17	14.87	13.66	0.43	0.13	0.19	0.24
1996	17.76	16.07	11.19	11.66	0.19	0.38	0.33	0.23
1997	17.17	14.63	13.45	11.85	0.19	0.28	0.29	0.29
1998	15.51	14.92	12.70	12.05	0.44	0.18	0.21	0.23
1999	16.74	16.05	14.60	13.20	0.24	0.16	0.22	0.24
2000	17.01	15.61	13.48	12.41	0.21	0.13	0.23	0.17
2001	16.80	15.41	14.54	13.75	0.24	0.16	0.33	0.24
2002	15.49	14.71	12.75	13.97	0.30	0.15	0.26	0.14
2003	15.71	14.25	13.63	13.55	0.51	0.15	0.36	0.21
2004	16.24	13.45	13.02	12.71	0.32	0.23	0.46	0.30
2005	16.44	13.95	11.08	12.62	0.26	0.23	0.35	0.15
2006	15.03	13.29	10.42	12.83	0.45	0.44	0.46	0.32
2007	14.32	12.65	10.06	12.24	0.94	0.41	0.52	0.38
2008	14.17	13.14	10.89	12.29	0.52	0.31	0.41	0.33
2009	15.46	12.56	10.08	12.54	0.38	0.36	0.52	0.32
2010	15.40	13.97	10.69	12.36	0.30	0.34	0.61	0.35
2011	15.25	15.09	12.54	13.92	0.40	0.23	0.33	0.34
2012	16.63	13.32	11.63	12.67	0.27	0.23	0.30	0.29
2013	15.50	13.48	9.95	12.43	0.20	0.25	0.51	0.31
2014	14.44	12.49	9.37	12.00	0.26	0.34	0.75	0.44
2015	13.17	8.07	7.97	9.99	0.33	1.05	1.01	0.39

Table S3.3: Delta smelt model parameter estimate summary. Maximum likelihood estimates (MLE), standard errors (SE), and lower (LCI) and upper (UCI) confidence intervals based on 95% confidence intervals based on likelihood ratio tests of profile likelihoods. Missing LCI or UCI entries denote values not estimable. Observation bias entries of exactly 1 denote fixed values.

	m_0				m_1				m_2			
Parameter	MLE	SE	LCI	UCI	MLE	SE	LCI	UCI	MLE	SE	LCI	UCI
$\log(N_{4,0})$	11.84	0.25	11.35	12.32	13.49	0.30	12.91	14.08	14.36	0.42	13.54	15.19
Recruitment												
$\beta_{R,0}$	3.63	0.17	3.29	3.97	2.75	0.16	2.44	3.07	4.81	1.09	3.60	
FebAdultLength	-0.53	0.21	-0.96	-0.10	-0.24	0.17	-0.59	0.11	0.03	0.19	-0.34	0.41
Outflow	-0.08	0.22	-0.53	0.36	-0.06	0.17	-0.41	0.29	-0.24	0.18	-0.59	0.13
Secchi	-0.47	0.23	-0.93	-0.01	0.09	0.19	-0.30	0.48	0.42	0.23	-0.03	0.88
Temperature	-0.07	0.25	-0.59	0.43	-0.26	0.21	-0.68	0.15	-0.34	0.21	-0.76	0.08
ACM	-0.31	0.28	-0.87	0.27	0.15	0.23	-0.31	0.62	0.46	0.24	-0.03	0.96
NJ	0.40	0.25	-0.11	0.90	-0.06	0.21	-0.50	0.36	-0.37	0.23	-0.84	0.08
$X2_{t-1}$	-0.07	0.17	-0.42	0.28	0.11	0.13	-0.16	0.38	0.15	0.13	-0.12	0.42
S_1 survival												
$\beta_{S_1,0}$	-1.37	0.21	-1.77	-0.90	-0.00	0.49	-0.84	1.24	-0.88	0.54		0.20
Outflow	0.73	0.33	0.17	1.59	1.23	0.53	0.30	2.47	1.10	0.52	0.08	2.22
Secchi	-0.25	0.22	-0.70	0.19	-0.49	0.30	-1.25	0.10	-0.38	0.24	-0.90	0.10
Temperature	-0.30	0.22	-0.76	0.13	-0.27	0.31	-0.96	0.35	-0.23	0.24	-0.75	0.25
S_2 survival												
$\beta_{S_2,0}$	-1.16	0.26	-1.68	-0.62	-0.52	0.45	-1.33	0.53	-2.20	1.24		-0.76
LSZ	0.01	0.28	-0.55	0.58	0.04	0.35	-0.72	0.77	0.05	0.24	-0.45	0.59
Secchi	0.16	0.32	-0.47	0.82	-0.34	0.41	-1.26	0.47	-0.25	0.28	-0.87	0.32
ACM	-0.23	0.27	-0.80	0.32	-0.13	0.35	-0.89	0.58	-0.02	0.24	-0.55	0.48
S_3 survival												
$\beta_{S_3,0}$	3.10	1.07	1.56	6.38	0.49	0.50	-0.31	1.88	-0.65	0.39	-1.32	0.44
OMR	0.67	0.67	-0.68	2.48	0.88	0.36	0.23	1.83	0.81	0.26	0.33	1.42
SouthSecchi	1.37	0.87	-0.16	3.91	0.66	0.41	-0.05	1.82	0.25	0.27	-0.24	0.99
ACM	-0.95	0.65	-2.62	0.36	-0.23	0.30	-0.94	0.40	0.06	0.23	-0.45	0.54
SBAge1Plus	-1.49	0.70	-3.44	-0.25	-0.52	0.33	-1.37	0.08	-0.10	0.25	-0.68	0.38
Process variance												
$\sigma_{p,R}$	0.68	0.13	0.47	1.00	0.48	0.11	0.29	0.75	0.45	0.11	0.26	0.71
σ_{p,S_1}	0.71	0.17	0.43	1.14	0.89	0.23	0.52	1.57	0.73	0.19	0.43	1.21
σ_{p,S_2}	1.16	0.19	0.85	1.65	1.41	0.29	0.97	2.18	1.01	0.24	0.65	1.60
σ_{p,S_3}	2.43	0.79	1.38	5.06	1.09	0.34	0.59	2.11	0.76	0.20	0.45	1.32
Observation bias												
ψ_{TMM}	1				1				0.08	0.09		0.31
ψ_{STN}	1				0.44	0.08	0.30	0.64	0.06	0.06		0.21
ψ_{MWT}	1				0.18	0.03	0.13	0.26	0.08	0.03	0.04	0.15
ψ_{SKT}	1				1				1			

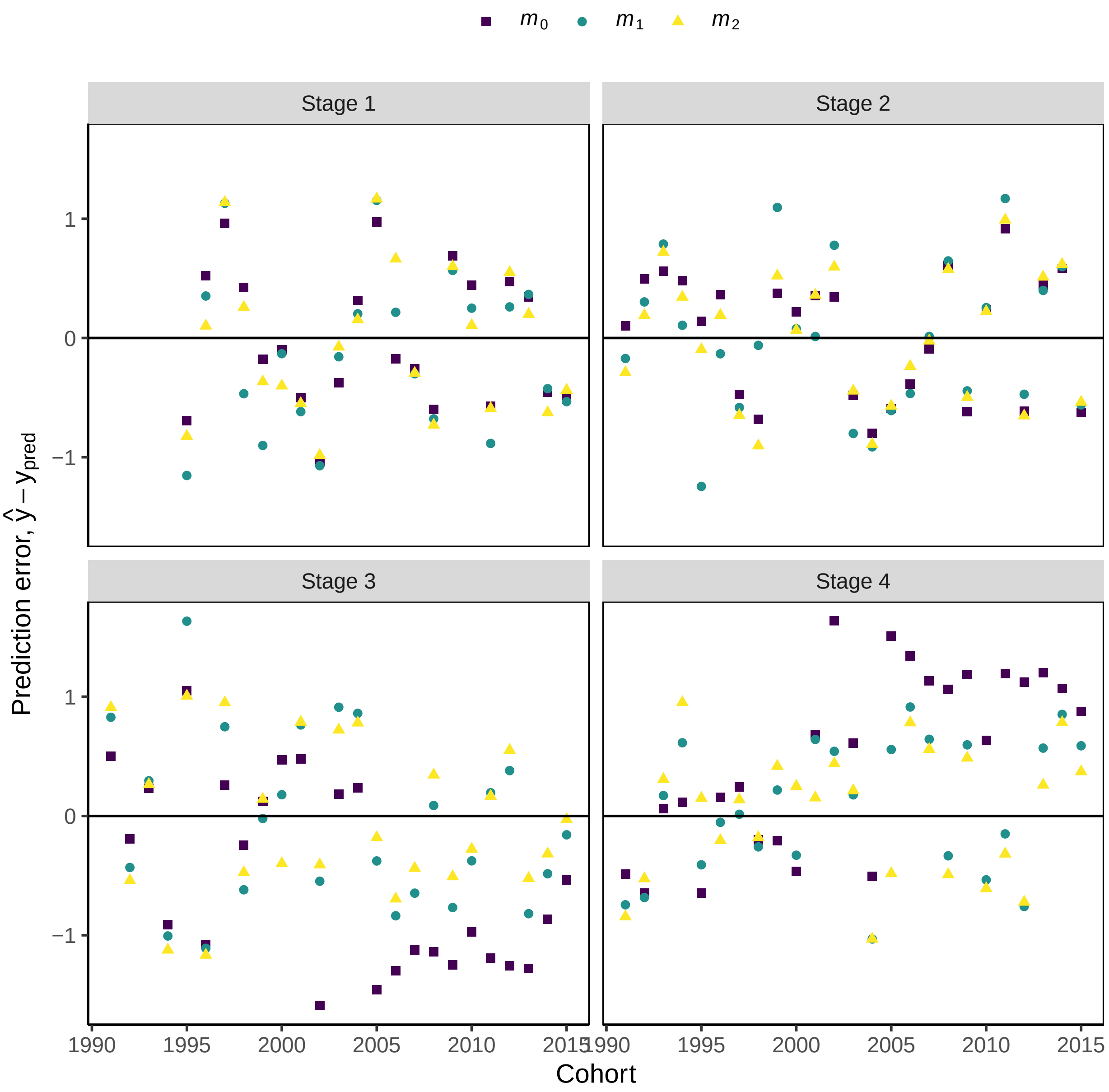

Figure S3.1: One-step ahead prediction residual times series plots by model and stage. Stage 1 residuals are available for the 1995-2015 cohorts, and all other stages have estimates for the 1991-2015 cohorts.

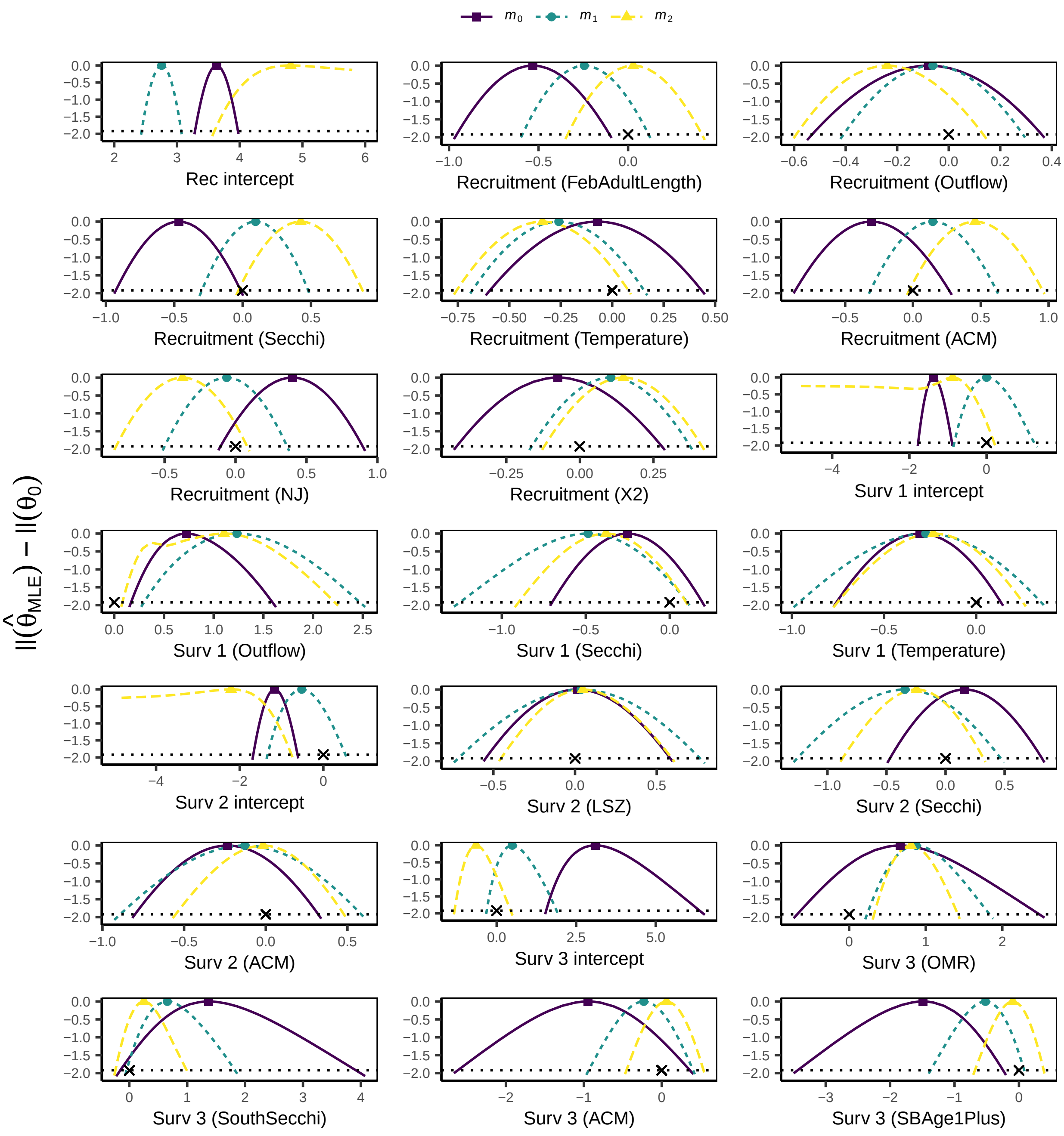

Figure S3.2: Delta smelt model profile log-likelihoods of recruitment and survival intercept and slope parameters. Values are shifted by the model maximum, $\ell(\theta_0) - \ell(\hat{\theta}_{MLE})$ to facilitate both comparison of changes across models and for likelihood ratio test statistic comparisons. Horizontal dotted lines are drawn at $\ell(\hat{\theta}) - 3.84/2$ corresponding to a 95% confidence interval of the likelihood ratio test. Points are at $(\hat{\theta}_{MLE}, 0)$. Black x's are at $(0, -1.92)$.

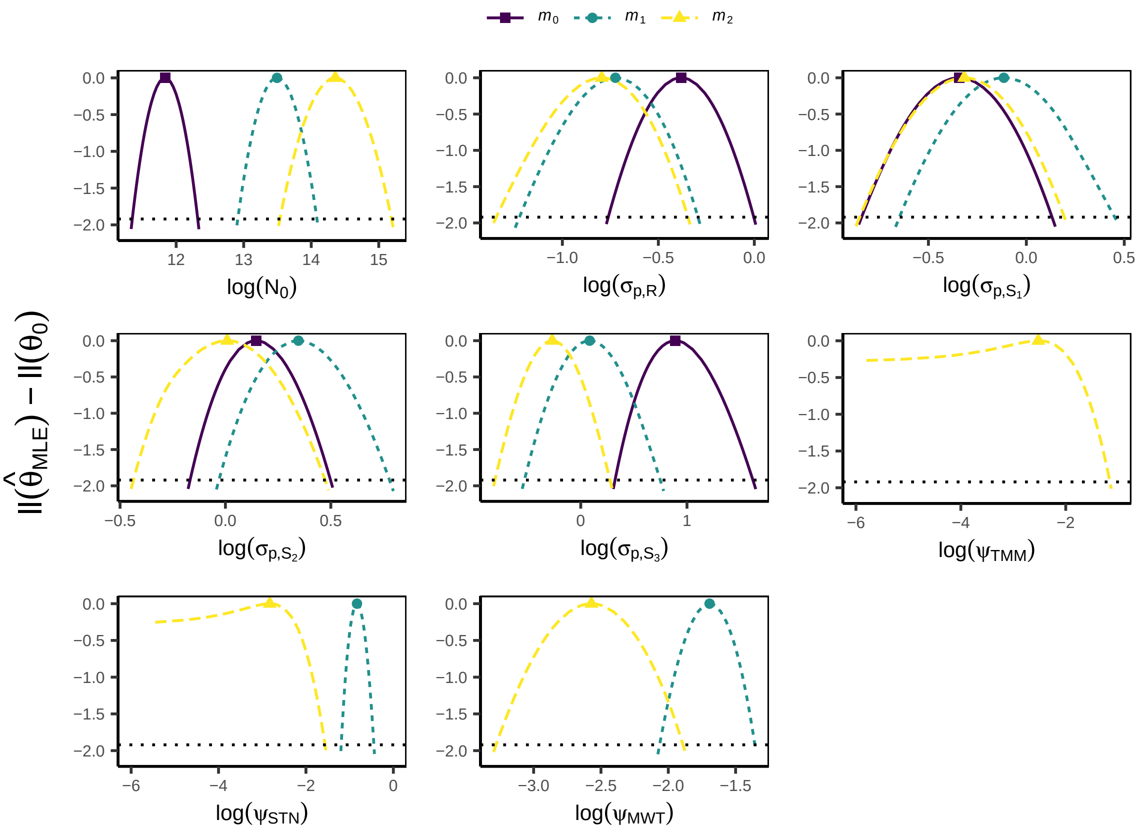

Figure S3.3: Delta smelt model profile log-likelihoods of the initial abundance, process variance, and observation bias parameters. Values are shifted by the model maximum, $\ell(\theta_0) - \ell(\hat{\theta}_{MLE})$ to facilitate both comparison of changes across models and for likelihood ratio test statistic comparisons. Horizontal dotted lines are drawn at $\ell(\hat{\theta}) - 3.84/2$ corresponding to a 95% confidence interval of the likelihood ratio test. Points are at $(\hat{\theta}_{MLE}, 0)$.

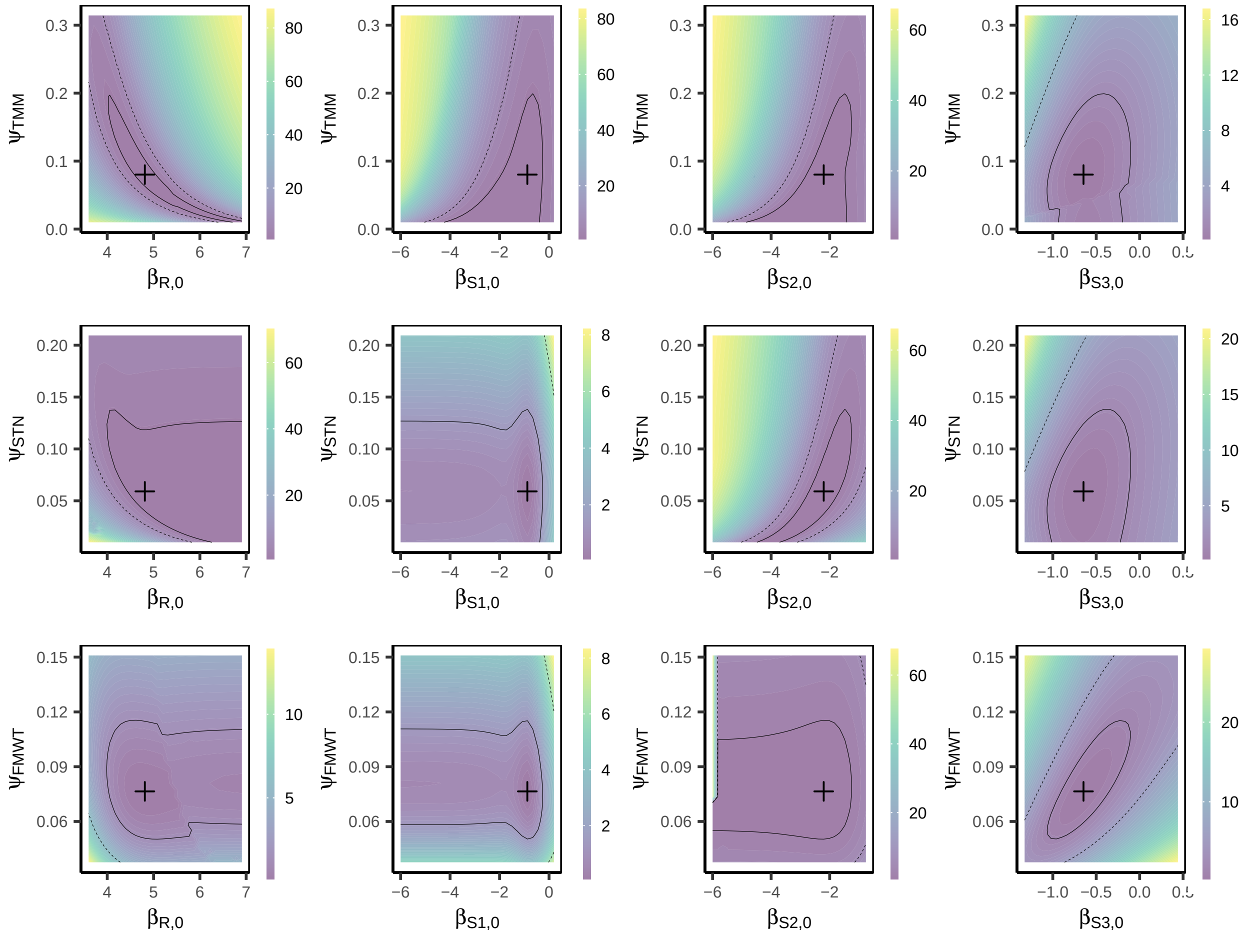

Figure S3.4: Delta smelt model joint profile log-likelihoods between the bias and intercept parameters of model m_2 (reference bias $\psi_{SKT} = 1$). Values are transformed to $-2(\ell(\theta_0) - \ell(\hat{\theta}_{MLE})$ to facilitate comparison with the likelihood ratio test. Contour levels are drawn at the 50% (solid) and 95% (dashed) confidence intervals. Cross is at the MLE location.

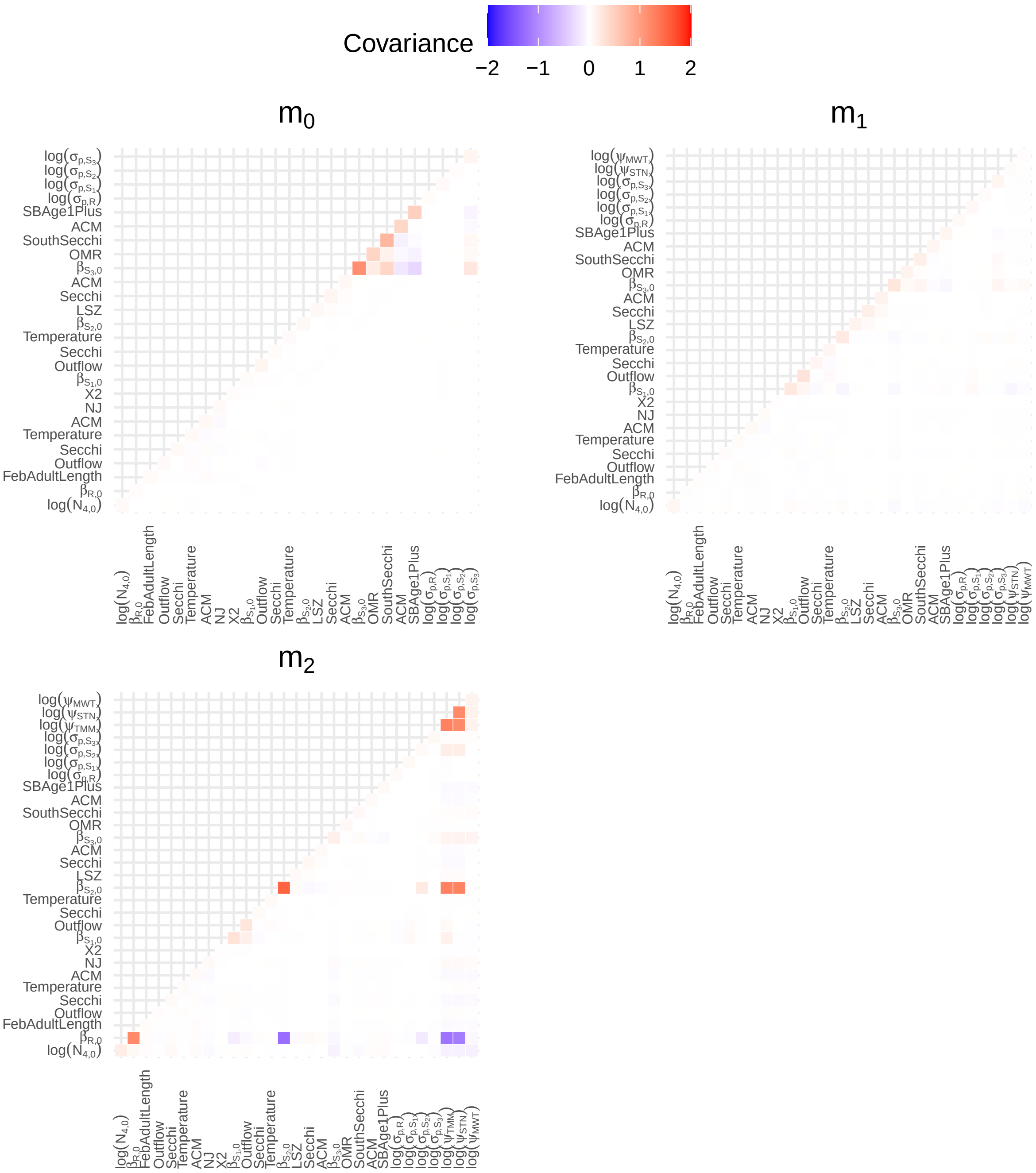

Figure S3.5: Delta smelt models fixed effect variance-covariance matrices as a heatmap.

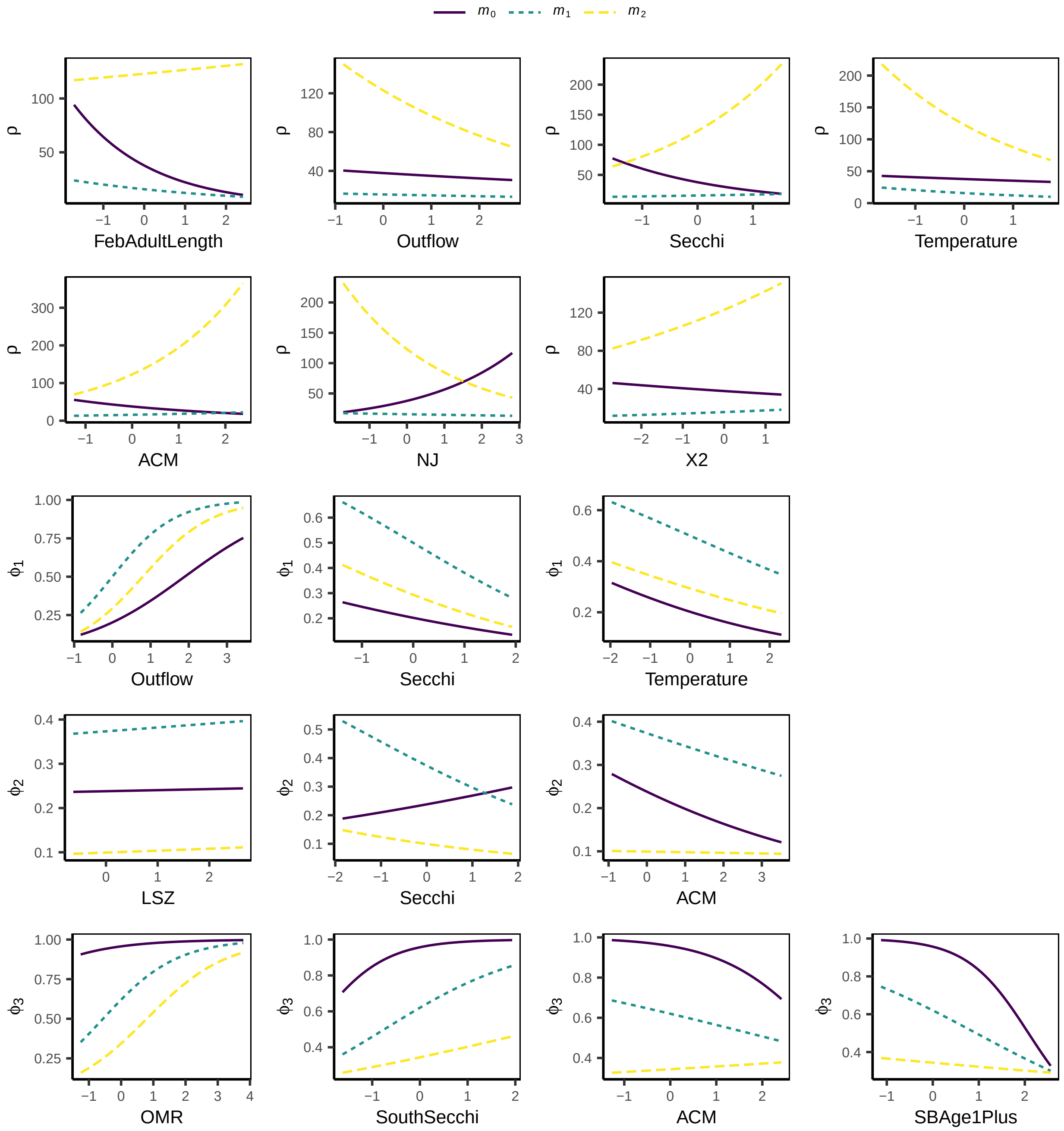

Figure S3.6: Delta smelt vital rate functions based on MLE parameter values.
